# DISC-3D: dual-hydrogel system enhances optical imaging and enables correlative mass spectrometry imaging of invading multicellular tumor spheroids

**DOI:** 10.1038/s41598-023-38699-1

**Published:** 2023-07-31

**Authors:** Rachel C. Avard, Megan L. Broad, Fereshteh Zandkarimi, Alexander J. Devanny, Joseph L. Hammer, Karen Yu, Asja Guzman, Laura J. Kaufman

**Affiliations:** 1 Department of Chemistry, Columbia University, New York, NY 10027 USA; 2 Department of Chemistry, Cardiff University, Cardiff, CF10 3AT Wales UK; 3 Department of Physics, Columbia University, New York, NY 10027 USA; 4 Department of Biological Sciences, Columbia University, New York, NY 10027 USA

**Keywords:** Mass spectrometry, Cancer models, Fluorescence imaging, Optical imaging, Biopolymers, Gels and hydrogels

## Abstract

Multicellular tumor spheroids embedded in collagen I matrices are common in vitro systems for the study of solid tumors that reflect the physiological environment and complexities of the in vivo environment. While collagen I environments are physiologically relevant and permissive of cell invasion, studying spheroids in such hydrogels presents challenges to key analytical assays and to a wide array of imaging modalities. While this is largely due to the thickness of the 3D hydrogels that in other samples can typically be overcome by sectioning, because of their highly porous nature, collagen I hydrogels are very challenging to section, especially in a manner that preserves the hydrogel network including cell invasion patterns. Here, we describe a novel method for preparing and cryosectioning invasive spheroids in a two-component (collagen I and gelatin) matrix, a technique we term dual-hydrogel in vitro spheroid cryosectioning of three-dimensional samples (DISC-3D). DISC-3D does not require cell fixation, preserves the architecture of invasive spheroids and their surroundings, eliminates imaging challenges, and allows for use of techniques that have infrequently been applied in three-dimensional spheroid analysis, including super-resolution microscopy and mass spectrometry imaging.

## Introduction

The in vitro study of cells cultured on flat, two-dimensional (2D) substrates has been employed for over a century and has been crucial in countless scientific discoveries. While 2D cell culture continues to be a standard method in molecular and cell biology, it has long been appreciated that it cannot recapitulate critical aspects of in vivo systems, including three-dimensional (3D) cell–cell and cell–environment interactions^1^. Such interactions are particularly important in the study of cellular processes that are explicitly emergent and multicellular; for example, in development and solid tumor growth and progression.

In recent years, 3D cell culture has been increasingly employed^2^. Here, multicellular entities are grown or prepared from cell lines or patient tissue—typically termed spheroids and organoids, respectively—and cultured in synthetic or natural hydrogels that mimic the in vivo extracellular matrix. Three-dimensional cell culture has been shown to better replicate physiology of human tissue such as gradients of nutrients and oxygen, differing proliferative zones, and cell–cell and cell–matrix interactions, providing clear rationale for using 3D culture to study biological questions that are multicellular in nature^3–6^. Among 3D hydrogels, collagen I environments show particular physiological relevance for many solid tumors. In breast cancer, high collagen I density is a known risk-factor for developing disease^7,8^ and particular density and arial organization of collagen fibers around tumors are associated with prognosis^9^. Interactions between cells and the collagen-rich stromal microenvironment are also important in pancreatic cancer^10,11^ and have been implicated in a variety of other cancers^12^. Collagen I is also an attractive environment for 3D cell culture as its biochemistry and network structure are supportive of efficient cell invasion. Moreover, it is an environment in which physical properties, such as fiber width, pore size and hydrogel stiffness can be readily tuned without altering biochemical composition, allowing study of the effects of these properties on cell invasive mode and efficiency^13–16^.

Despite the fact that it is well established that 3D cell culture, and collagen I hydrogels in particular, provide a high degree of physiological relevance and opportunities to disentangle the importance of biochemistry from physical properties on cell behavior, adoption of 3D cell culture has been relatively slow. Reluctance stems, in part, from the challenges associated with acquiring high-resolution optical microscopy images in such environments^6^. Cells sense and respond to environmental stiffness over distances of at least tens of, and arguably well over a hundred, microns^17^. As such, for cells to behave as they would in an isotropic 3D environment, they must be positioned well above the stiff imaging substrates. This may result in cells being beyond the working distance of typical high numerical aperture objectives, high background signal due to scatter from outside the imaging plane, and auto-fluorescence from cells or the hydrogel surrounding cells. Moreover, the 3D environment may hamper diffusion of small molecules or antibodies through the sample inhibiting both drug studies and introduction of fluorescent labels in these contexts. Challenges associated with 3D samples can be especially acute in the most information-rich approaches such as super-resolution optical imaging, where high signal to noise is paramount, correlative approaches in which multiple modalities are employed, and non-optical approaches such as mass spectrometry imaging where scattering from thick samples precludes data collection except at the sample surface.

As in tissue, sectioning can be used on 3D hydrogel samples to alleviate these issues. Unfortunately, some of the same properties that make collagen I permissive of cell invasion also make it poorly suited to sectioning. In particular, collagen I gels are weak, with storage moduli typically 10–100 Pa for preparations used for cell invasion experiments^13,14^. Together with the microporous nature of collagen I hydrogel networks, this makes the gels particularly likely to collapse upon sectioning. Demonstration of sectioning cell-bearing 3D collagen I hydrogels is rare^18,19^, though sectioning of spheroid cores (lacking an invasion-competent matrix) can be achieved^20–23^. While informative, investigation of spheroid cores alone precludes investigation into cell migratory patterns, cell interactions with extracellular matrix proteins, and the metastatic cascades associated with cancer cell migration. Beyond this, typical embedding agents can present challenges for particular experiments. Paraffin embedding affects protein antigenicity, inhibiting antibody-based labeling^24,25^ while also requiring sample dehydration that disrupts hydrogel network structure. Embedding in optical cutting temperature compound (OCT) results in readily sectioned samples^19,26^ but is incompatible with mass spectrometry experiments, as one of OCT’s main components, polyethylene glycol, produces ions that compete with endogenous small molecule ions, resulting in a significant decrease in spectrum intensity and quality^27,28^. Additionally, the sectioning methods that have been used previously on cells with an invasion-competent matrix^18,19^ have relied on cellular fixation, which interferes with a variety of analytical approaches, including mass spectrometry imaging of lipids and related lipidomics analyses, as fixation protocols wash away several small metabolites and lipids^29–31^.

Here, we describe a novel method of preparing and cryosectioning spheroids in a two-component—collagen I and gelatin—matrix, a technique we term dual-hydrogel in vitro spheroid cryosectioning of three-dimensional samples (DISC-3D). This technique takes advantage of the fact that gelation of collagen I occurs upon increasing temperature while that of gelatin occurs with decreasing temperature, allowing for serial temperature-controlled gelation of the interpenetrating networks. The DISC-3D protocol is supportive of cellular proliferation and invasion, and individual cell and surrounding extracellular matrix integrity is preserved during sectioning. Moreover, the protocol does not interfere with protein antigenicity and does not require fixation (though is compatible with it), allowing for investigation into lipid distributions in invasive cells. As such, in addition to allowing for an array of optical microscopies including super-resolution microscopies, the DISC-3D protocol allows for mass spectrometry imaging of invasive spheroids.

## Results

### DISC-3D protocol

**Figure 1:**
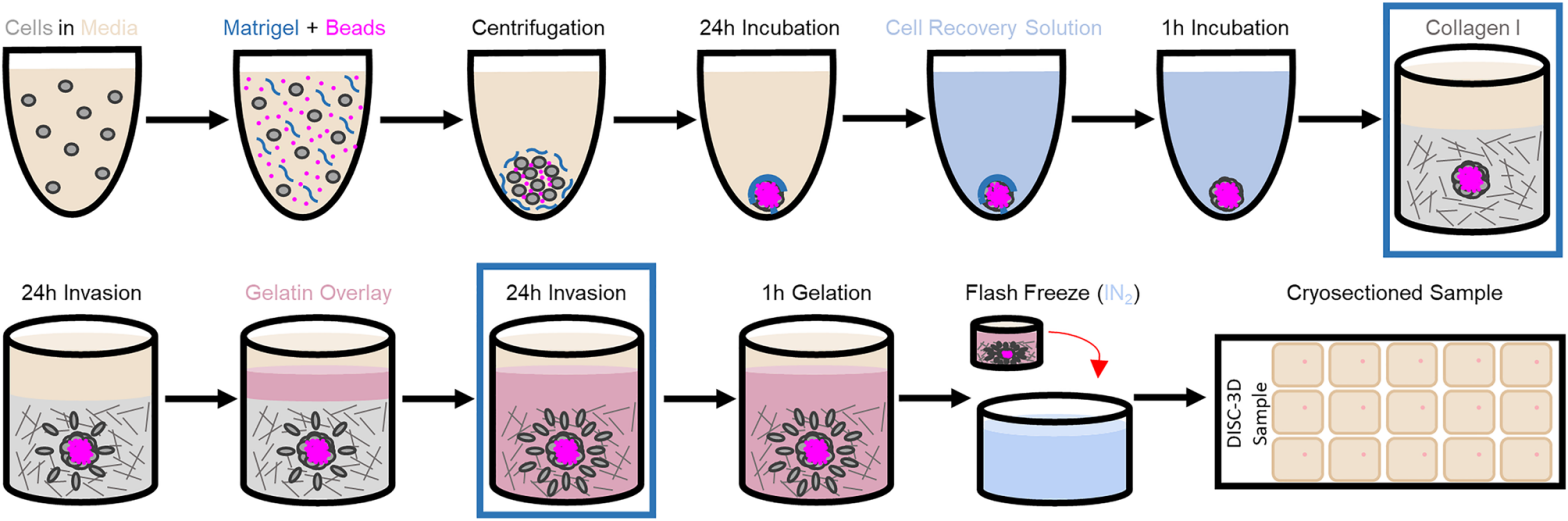
DISC-3D Protocol. Spheroids are formed in the presence of fluorescently labeled polystyrene beads, which provide a visual cue for locating spheroids in later steps. Spheroids are placed in collagen I solutions that are allowed to gel. Following spheroid implantation and initial cell invasion, the samples are overlaid with warmed gelatin. The gelatin is allowed to diffuse through the sample for 24 h as the cells continue to invade, and is then gelled for 1 h at 4 °C prior to flash freezing. The sample then can be cryosectioned and imaged via a variety of techniques. Variations of the procedure optimized for distinct experiments are described in Methods and in Fig. S1. A representative spheroid subjected to the DISC-3D protocol and imaged via brightfield microscopy is shown in Fig. S2b,c at the two points in the protocol outlined in blue boxes.

As is schematically depicted in Fig. 1 and Fig. S1, the DISC-3D protocol consists of forming a spheroid, placing it in a collagen I solution that is gelled around the spheroid, allowing the spheroid to invade, and overlaying it with a gelatin solution that diffuses through the sample in advance of gelatin gelation. This creates a dual, interpenetrating collagen I/gelatin hydrogel network around the spheroid. At this point, samples are ready for freezing and cryosectioning.

The combination of collagen I and gelatin is particularly appealing for several reasons. First, gelatin is denatured collagen, assuring that the cells are exposed to among the simplest biochemical environments possible in such an approach. Critically, while collagen goes through a sol–gel transition upon heating, gelatin does so upon cooling. This allows serial self-assembly of the two networks with temperature changes. Moreover, gelatin is found to not impact cell invasion while it is a solution (Fig. S2), so the timing of the addition of gelatin and the time required for its diffusion through the sample does not inhibit the study of invasion and invaded cells. Finally, the DISC-3D protocol addresses the need for a bi-functional scaffold with two distinct pore sizes. While collagen I self-assembles into fibers with pore size on the order of microns, a network structure highly supportive of cellular invasion, gelatin forms a nanoporous network. The nanoporous gelatin provides the matrix with physical properties suitable for cryosectioning unlike a (single component) highly porous collagen I hydrogel. With cryosectioning of spheroids, including invasive spheroids, possible, the DISC-3D protocol enables a number of experiments not previously feasible for such samples. While Fig. 1 shows one implementation of the DISC-3D protocol, the protocol can be adapted to fit different experimental needs, examples of which are described in Methods and schematically depicted in Fig. S1.

### Confocal microscopy and immunocytochemistry of DISC-3D samples

**Figure 2:**
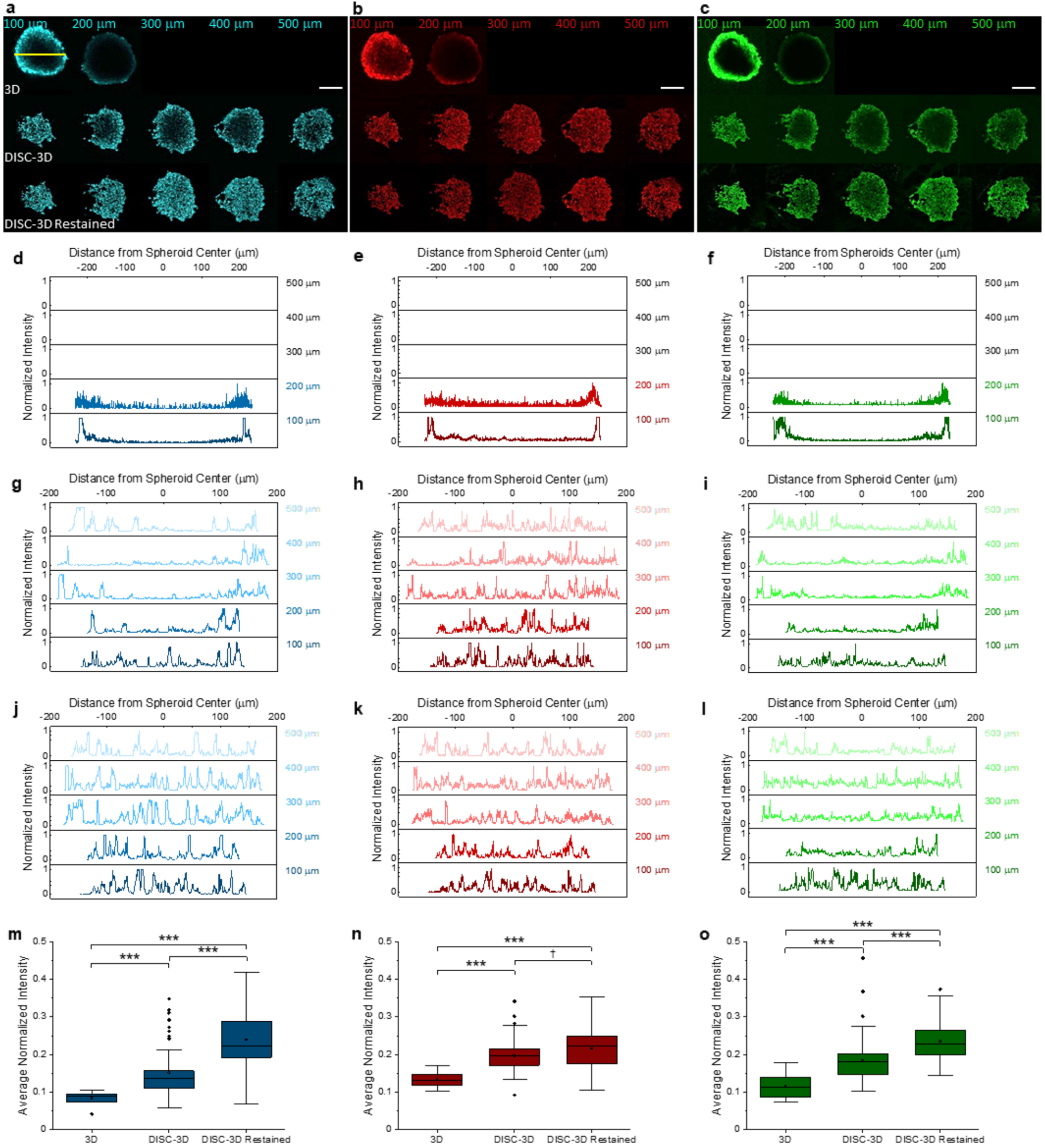
Image quality comparison in lateral and axial dimensions. All data here was obtained from the same MDA-MB-231 spheroid, which was prepared following the DISC-3D protocol outlined in Fig. S1b, and fluorescently labeled with DAPI (blue), phalloidin (red), and anti-β1 integrin antibody (green). (a-c) Spheroid images collected (top row) with confocal fluorescence microscopy in 3D, (middle row) following the DISC-3D protocol including cryosectioning (10 μm slices), and (bottom row) following re-staining after the DISC-3D protocol. Labels indicate depth into the spheroid at which the image was collected. The top row of a-c only shows slices at 100 and 200 μm, as the working distance of the objective precluded imaging deeper into the sample. Scale bar = 200 μm. (**d–j**) Intensity profiles of a linear ROI drawn from one edge of the spheroid to the other [yellow line in the spheroid at top left in (**a**)] for all images shown in (**a–c**), with 3D images represented in (**d–f**), DISC-3D cryosectioned data represented in (**g–i**), and DISC-3D re-stained data represented in (**j–l**). Intensity is normalized to the maximum intensity in that intensity profile. (**m–o**) Signal averaged across normalized intensity profiles and slices across the spheroid in 3D, DISC-3D, and DISC-3D re-stained samples for (**m**) DAPI, (**n**) phalloidin, and (**o**) β1 integrin respectively. We note that such a comparison underemphasizes the difference between traditional 3D and DISC-3D images, as the traditional 3D images are only assessed 100 and 200 μm into the sample. *p < 0.05, **p < 0.01, ***p < 0.001, ^†^p > 0.05 (n = 12, 59, and 46 slices for 3D, DISC-3D, and DISC-3D re-stained, respectively).

The increased complexity of 3D culture poses unique challenges for sensitive detection and quantification of cellular targets of interest. In such a crowded environment, detection of target antigens may be limited by probe diffusivity through dense regions of cells and the surrounding extracellular matrix as well as by loss of signal due to light scattering from thicker samples. While traditional embedded sectioning can alleviate this issue, no extant techniques are suitable for the full array of optical and mass spectrometry imaging experiments we wish to perform. Among other things, the DISC-3D protocol takes advantage of the superior antigen preservation of cryosectioning methods over paraffin-based approaches to facilitate high-content, high resolution imaging techniques that are typically challenging in 3D samples. This is illustrated first in Fig. 2, which shows a single MDA-MB-231 breast cancer spheroid prepared using the DISC-3D protocol and (prior to invasion) fixed and dyed with phalloidin—AlexaFluor567 (a small, fluorescently conjugated molecule that binds to cellular f-actin), DAPI (a small fluorescent molecule that intercalates into DNA), and a directly conjugated anti-β1 integrin antibody (clone P5D2) labeled with AlexaFluor488. The work flow associated with this experiment is shown in Fig. S1b. Optical images of this spheroid were first obtained prior to the addition of gelatin and cryosectioning (Fig. 2a–c, top row) to allow for comparison of image quality before and after sectioning. Images along the axial dimension of the intact spheroid were taken every 10 μm, and representative images of the spheroid every 100 μm are shown. These images (Fig. 2a–c, top row) and associated intensity profiles obtained from linear ROIs drawn across the center of each spheroid core (Fig. 2d–f) reveal that while all three of the fluorophores can be visualized at the spheroid periphery with confocal fluorescence microscopy, none are seen in the spheroid core. This is particularly noteworthy in the case of DAPI, which should have uniform distribution through the spheroid. The apparent localization of DAPI to the spheroid periphery indicates limited diffusion of the dye through the spheroid and/or signal loss due to inefficient excitation and/or collection of fluorescence from the spheroid core. In an attempt to combat these difficulties, this spheroid was taken through the final steps of the DISC-3D protocol, resulting in sections that were 10 μm thick. Imaging following sectioning reveals fluorescence through the full axial dimension of the spheroid as well as across the radial dimension, though enhanced intensity is still observed at the spheroid periphery for several of the dyes, as is particularly apparent in slices obtained from deep within the spheroid. These observations suggest that the lack of interior signal in 3D samples imaged via confocal microscopy can be partially attributed to fluorescent signal loss due to light scattering but is also due in part to poor penetration of the fluorescent labels through the dense 3D sample. As such, this same spheroid, which had already been through the full DISC-3D procedure including cryosectioning, was re-stained with each of these dyes and imaged (Fig. 2a–c, bottom row). These images show relatively uniform staining of DAPI across the spheroid in the xy plane for all slices (Fig. 2j). Thus, it can be concluded that confocal fluorescence images obtained from the original spheroid were impacted by limitations in imaging depth, scattering of excitation and/or emission in the sample, and sample permeability and dye diffusion. Images of labeled phalloidin in the re-stained samples also show a relatively uniform staining throughout the spheroid (Fig. 2k), consistent with the expectation that F-actin is present in similar quantities in all cells. Figure 2n shows that there is no statistical difference between the initial DISC-3D phalloidin images and the re-stained DISC-3D phalloidin images, suggesting phalloidin—unlike DAPI—did fully penetrate the sample in the 3D context.

Antibody-based probes suffer from similar sample penetration issues as their small molecule counterparts. The staining of β1 integrin demonstrates a strong accumulation of this protein at the spheroid periphery in 3D and DISC-3D images (Fig. 2c, top two rows), indicative of poor antibody penetration. Although one might expect high concentrations of β1 integrin at the collagen-spheroid interface, prior work has demonstrated that β1 integrins also are involved in extracellular matrix-mediated cell–cell adhesions throughout the spheroid interior^32,33^. Indeed, re-staining of DISC-3D samples reveals this to be true, with β1 integrin found throughout the spheroid core (Fig. 2c, bottom row; Fig. 2l, o). The set of results shown in Fig. 2 demonstrates how the DISC-3D protocol, especially when coupled with staining after sectioning, can reveal critical but otherwise obscured details of molecular distribution in a sample that could be incorrectly interpreted if approached only via staining and/or imaging in 3D. Additionally, given the differences in probe penetration efficiency observed here (in which poor penetration is apparently not exclusive to bulkier probes), it is clear that it can be difficult to predict situations where dye penetration issues will occur, highlighting the utility of the DISC-3D protocol followed by staining to correctly identify molecular distribution in 3D samples.

In addition to improving image quality of fluorescently labeled 3D samples, the DISC-3D protocol facilitates use of fluorescent labelling strategies that are impractical or impossible to carry out in typical 3D in vitro samples due to limited signal, low signal to background, and/or poor sample penetrability. Many directly-conjugated antibodies and even the occasional small molecule fluorophore label fall into this group; additionally, secondary antibody labeling nearly always fails in spheroids cultured in 3D hydrogels. While there are some reports of use of secondary antibodies in sectioned 3D samples, the methods employed require dehydration, fixation, and embedding steps that do not typically preserve the integrity of the invasive front of a spheroid^34,35^.

**Figure 3:**
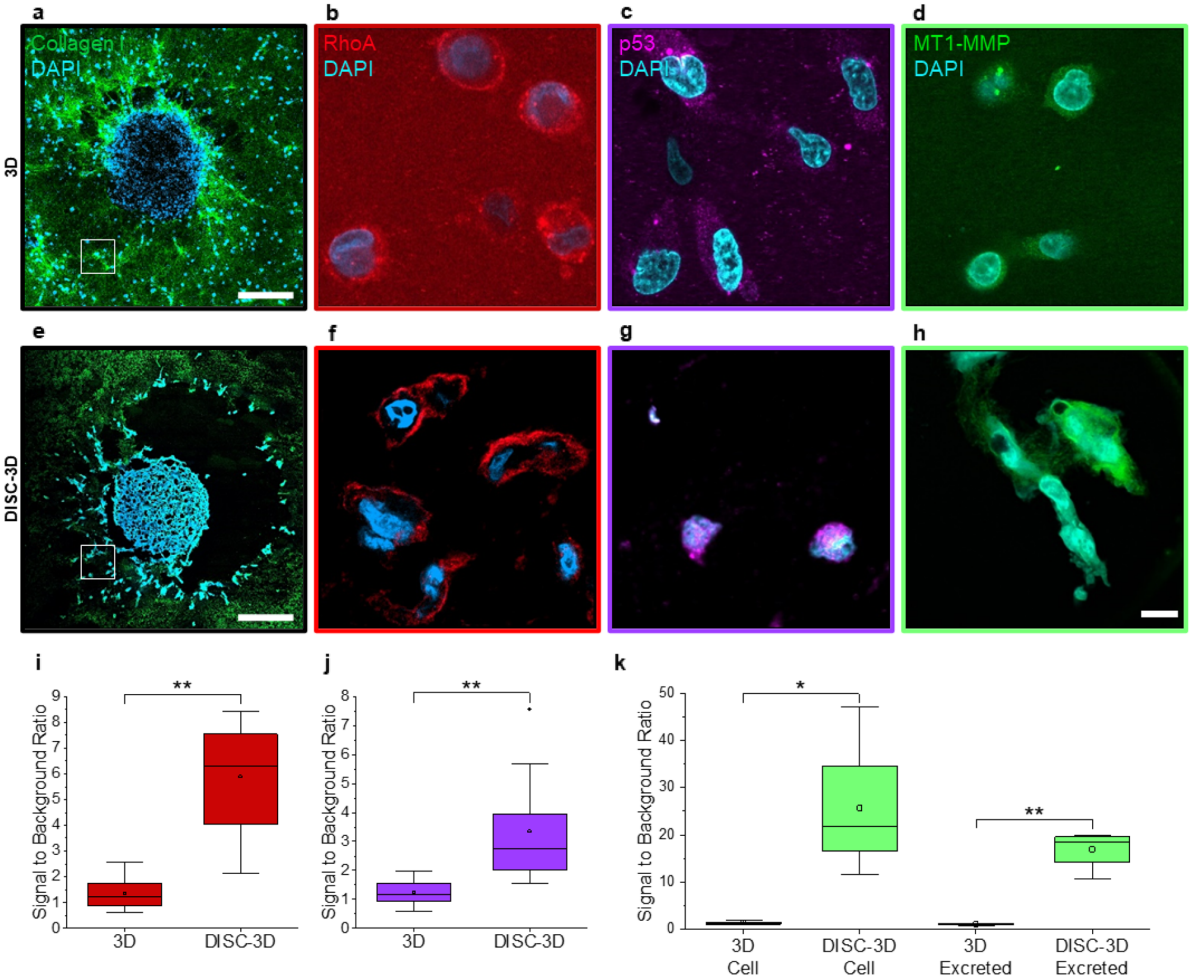
DISC-3D enhances resolution and accurate localization. (**a–h**) Invasive MDA-MB-231 spheroids imaged via confocal fluorescence microscopy (**a–d**) in 3D or (**e–h**) following DISC-3D preparation (10 μm slices). (**a,e**) Exemplary spheroids labeled with DAPI (cyan) and with collagen I also fluorescently labeled (green). Images of individual cells at the leading edge of the invasive spheroid front were acquired, as depicted in the white boxes as well as in panels (**b–d**) and (**f–h**). (**b–d, f–h**) Representative invading cells from spheroids labeled with DAPI (cyan) and (**b,f**) anti-RhoA (red, secondary), (**c,g**) anti-p53 (magenta, secondary), or (**d,h**) anti-MT1-MMP (green, directly conjugated) antibodies. In all cases, images of individual cells at the leading edge of the invasive spheroid front were acquired, such as those highlighted by the white boxes in (**a,e**). (**a,e**) Standard confocal microscopy images; (**b–d, f–h**) are Airyscan images. (**i–k**) Signal to background ratio of the 3D and DISC-3D samples labeled for (**i**) RhoA, (**j**) p53, and (**k**) MT1-MMP. For MT1-MMP, signal to background was assessed both on the cell membrane and in the immediate environment around the cell, where MT1-MMP is excreted. Scale bar = 200 μm in (**a,e**) and 10 μm in (**b–d, f–h**); *p < 0.05, **p < 0.01, ***p < 0.001, ^†^p > 0.05.

Here, spheroids were stained with three dyes that are regularly used in 2D cell culture but have not been used successfully in our laboratory on 3D spheroid samples and for which we find no evidence in the literature of successful labeling in 3D samples supportive of cellular invasion. Specifically, we attempted to visualize MT1-MMP, RhoA and p53 through directly conjugated antibodies (MT1-MMP) or a combination of primary and fluorescently labeled secondary antibodies (RhoA and p53) in cells at the invasive front of a spheroid. For MT1-MMP, the chief difficulty is presumably the low levels of target protein present and its location near or in the extracellular environment, where background signal can be particularly high. For RhoA and p53, secondary labeling was attempted. Secondary labeling is a convenient approach in cases where directly conjugated antibodies are not commercially available and affords valuable signal amplification for low-abundance targets. Here, the primary challenge is the two-step nature of secondary labeling that requires effective serial penetration of two large molecules through the sample. As Fig. 3b–d demonstrates, images obtained using these dyes in 3D approaches show low signal to background while those obtained via staining after sectioning using the DISC-3D protocol have significantly higher signal to background (Fig. 3f–k). We note also that invasive cell phenotype is maintained in DISC-3D samples, something that to the best of our knowledge has only been demonstrated once previously for invasive cells in a 3D hydrogel following sectioning^19^.

Importantly, confocal microscopy of DISC-3D spheroids shows molecular distributions that are consistent with those reported in the literature, while confocal images of intact 3D samples do not. For example, RhoA must be anchored to the cell membrane in order to be activated and, as such, is expected to be localized primarily to the membrane, particularly in invading cells^36,37^. Due to the poor signal to background in cells stained in the 3D hydrogel and imaged with a traditional confocal approach, RhoA intensity is barely above background (Fig. 3d), and localization of the fluorophore is difficult to determine from these images. In contrast, invasive cells visualized following the DISC-3D protocol show clear localization of RhoA to the cell membrane (Fig. 3f). Likewise, p53 binds directly to DNA and is thus expected to be localized primarily in the cell nucleus, as has been routinely demonstrated in 2D samples^38^. The traditional 3D image fails to show this localization (Fig. 3c), while DISC-3D samples show the protein confined to the nucleus (Fig. 3g). Finally, MT1-MMP exists as both a transmembrane as well as an excreted, soluble protein^39,40^. In the traditional 3D image, no protein can be seen in the extracellular environment, and even localization of the dye within the cell is challenging to determine given the low signal to background ratio (Fig. 3d). In contrast, MT1-MMP localization to the membrane and in the local surroundings of invasive cells is evident in samples prepared via the DISC-3D protocol (Fig. 3h).

Given the potential to stain and image DISC-3D samples following cryosectioning, we also assessed the time period over which staining and imaging could be performed with high fidelity. To assess sample longevity, we prepared a spheroid via fixation, freezing, cryosectioning and staining. The sample was then placed in an airtight bag with desiccant for over 10 months at 4 °C. Following the storage period, the sample was placed in a desiccator at room temperature for 1 h and then imaged (Fig. S3). The sample showed no apparent degradation, and signal intensity and fluorophore localization was as expected. Further, following the 10-month storage period, an additional dye (phalloidin) was added to the sample to assess whether labeling following long term storage could be achieved. Figure S3 shows that such labeling is adequate for visualizing actin in the sample, indicating long term preservation of cellular and sub-cellular structure in samples prepared in this manner. The ability to stain samples after the DISC-3D protocol and long-term storage allows for follow-up sample interrogation after, for example, development of new hypotheses and identification of new labeling targets.

### Super-resolution imaging of collagen architecture in DISC-3D samples

An essential component of the DISC-3D method is the preservation of the basic architecture of the scaffold in which the cells are embedded. Although control experiments demonstrate that the gelatin-collagen interpenetrating network is supportive of cellular invasion in a manner comparable to pure collagen I gels (Fig. S2), we further probed the structure of DISC-3D sectioned hydrogels via optical microscopy to confirm that the collagen fiber network, whose fibrillar, microporous structure is critical to cellular invasion, remained intact after sample processing and sectioning. To simplify analysis of the fibrillar network, we employed bare (spheroid free) hydrogels composed of collagen I and gelatin, prepared in accordance with the DISC-3D protocol. Beyond validating network structure, these measurements also provided a convenient testbed for evaluation of image quality of 3D and DISC-3D samples across a variety of optical microscopy techniques, as collagen fibers formed under the conditions used here have rather uniform diameter of 50–100 nm^15^.

**Figure 4:**
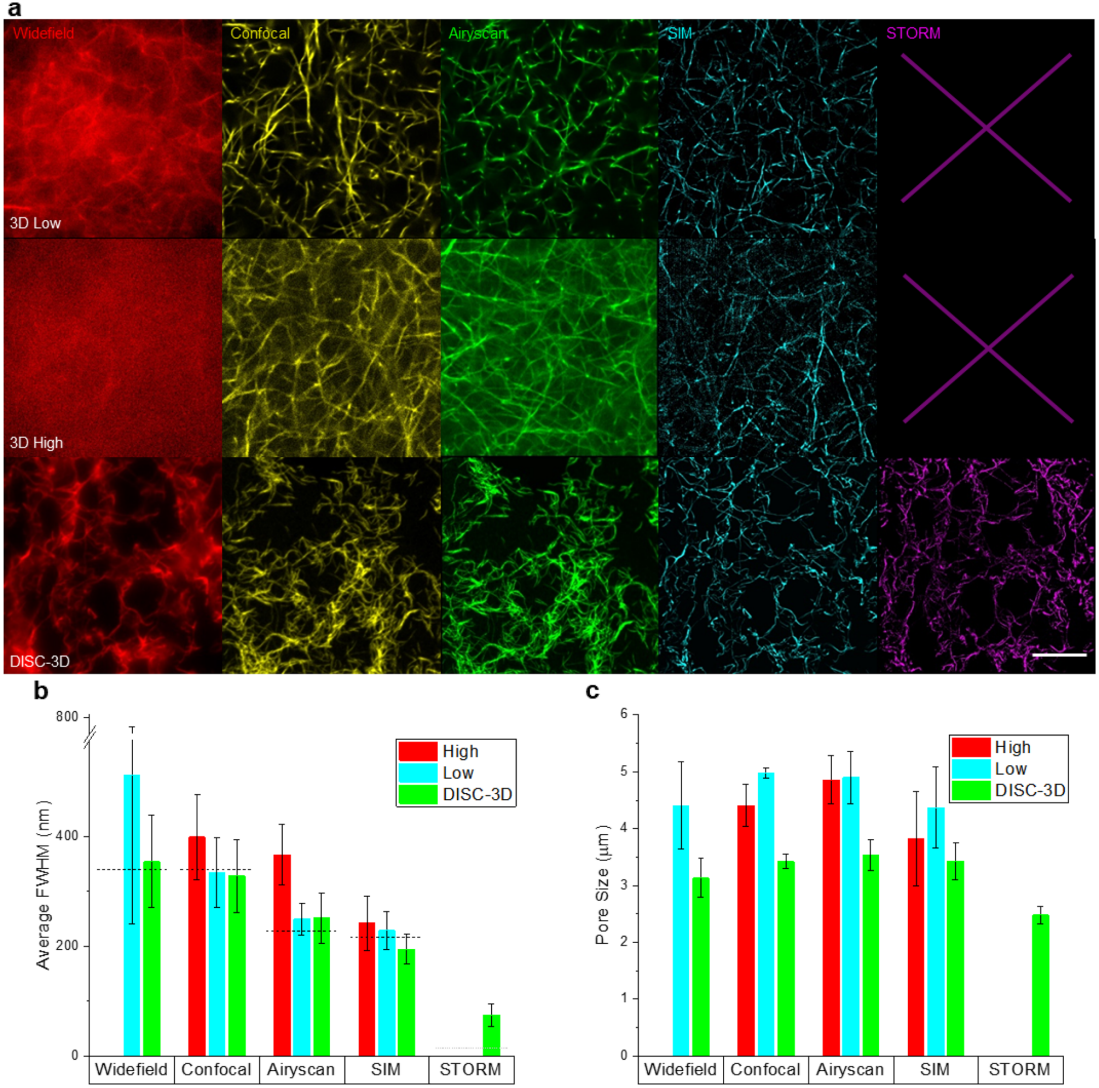
(**a**) Collagen I images taken (top) in 3D ≈ 10 μm above the coverslip [3D low], (middle) in 3D > 100 μm into the gel [3D high], and (bottom) following DISC-3D preparation (4 μm slices). From left to right, the columns show images taken using widefield microscopy, confocal microscopy, Airyscan imaging, lattice SIM, and STORM in a HILO implementation. No image is presented for 3D STORM imaging in the top two rows because (3D low) filtering out-of-plane light was insufficient or (3D high) the technique could not be implemented at such depths. Scale bar = 20 μm. (**b**) Mean measured full width at half maximum (FWHM) of collagen fibers from each microscopy technique. Error bars show standard deviation. Expected minimum FWHM of each approach is shown via dashed horizontal lines. (**c**) Pore size of collagen networks determined across imaging techniques and sample preparations (see Methods for details). Error bars show standard deviation. Statistical significance, number of samples assessed, and additional information about resolution of each technique are provided in Fig. S4.

Figure 4a shows representative images of collagen using five fluorescence microscopy techniques: widefield, confocal microscopy, Airyscan microscopy, lattice structured illumination microscopy (SIM), and stochastic optical reconstruction microscopy (STORM). The latter three approaches are considered super-resolution techniques: Airyscan imaging enhances resolution via a multi-element detector and deconvolution, lattice SIM recovers high-resolution information through the use of a diffraction grating, and STORM is a localization-based super-resolution technique that enhances resolution through sequential imaging of individual fluorophores^41–43^. STORM microscopy was conducted with highly inclined and laminated optical sheet (HILO) illumination, a variation of total internal reflection microscopy that increases penetration depth^44^.

For each technique, images of collagen fibers were collected near the substrate (10–25 μm into the gel, referred to as 3D low, top row), well above the substrate (> 100 μm into the gel, referred to as 3D high, middle row), and following DISC-3D preparation and cryosectioning (bottom row). All gels investigated were subjected to the first portion of the DISC-3D protocol, the addition and gelation of gelatin. Thus, the only difference between the samples in the DISC-3D images (bottom row) and the other samples (top and middle rows) is that the DISC-3D samples were frozen, removed from the sample wells, and sectioned. First, we note that qualitatively, images of the hydrogel collected near the coverslip appear similar to images of DISC-3D samples across techniques, suggesting that hydrogel structure is not adversely affected by the DISC-3D sample preparation procedure. Generally, DISC-3D samples show enhancements in image quality for all microscopy techniques explored. Widefield microscopy, which suffers from out of plane signal for 3D samples, shows obvious gains in image quality for DISC-3D samples relative to intact 3D samples (Fig. 4a, leftmost column). Confocal microscopy, Airyscan, and SIM, while providing excellent image quality and revealing consistent fiber morphology low in the gel, also show declines in image quality at increased imaging depths (Fig. 4a). We note that imaging at such depths assures the cells interrogated do not sense the underlying stiff substrate; therefore, maximizing image quality in this region is critical for studying cell behavior in a physiologically relevant context. STORM images were poor, and fibers were difficult to discern both low and high in the 3D gels, ostensibly due to challenges filtering out of plane emission in this highly scattering sample, as such filtering is critical to identification and localization of single fluorophores. The enhancement in image quality observed for DISC-3D samples opens the door to revealing details about system microstructure that would not otherwise be accessible in traditional 3D culture contexts.

Indeed, following such qualitative assessment, we quantified both apparent fiber width and pore size across sample preparations and techniques, as these are the key network structural properties of collagen I hydrogels^45^. Apparent fiber width was determined from the full width at half maximum (FWHM) of Gaussian fits to intensity profiles across fibers, considering only fibers that were well fit by this method (R^2^ > 0.95, Fig. S4a). For most microscopy techniques explored here, we find fiber widths larger than the expected fiber thickness of 50–100 nm, consistent with the measured resolutions of these techniques (Fig. 4, Fig. S4c). Given that the true fiber width falls below measured resolutions for widefield, confocal, Airyscan, and SIM, only STORM imaging—which can only be applied to DISC-3D samples—can accurately report fiber thickness (Fig. 4b). Here, we find a fiber width of approximately 75 nm in DISC-3D samples, in good agreement with measurements by electron microscopy and atomic force microscopy^46^.

Next, we assessed the pore sizes of hydrogels across imaging methods and sample preparations, as described in Methods. For 3D samples, measured pore size was similar in all imaging contexts where imaging was successful, returning a value of ≈ 4 μm (Fig. 4c). This pore size is similar to that reported previously from fluorescence microscopy^47^ and somewhat smaller than that previously reported for collagen I gels of this concentration and biological source^13^; however, those measurements were performed via confocal reflectance microscopy, which is insensitive to a subset of fibers^48^, yielding an apparently less dense network. DISC-3D prepared samples yielded somewhat smaller pore sizes than their 3D gel counterparts when analyzed in the same manner, with pore size ≈ 3 μm (Fig. 4c). The differences in pore size distribution and the fact that the fibers look somewhat more curved and entangled in the DISC-3D images than in other images may reflect a small degree of collapse associated with the sample manipulation required to prepare the sample for sectioning and/or the sectioning itself. In particular, we found no changes to the sample structure occur upon freezing, but small changes do occur upon removing the silicone spacers surrounding the sample, which must be done before cryosectioning (Fig. S5). Removing the silicone spacers may perturb anchoring points of the sample-spanning collagen network, leading to a small degree of gel contraction and collapse. Additional perturbation may occur during sectioning. Despite these small differences, DISC-3D samples clearly maintain the microporous structure of 3D collagen networks, providing confidence that detailed, high-resolution imaging of cell-extracellular matrix interactions and changes in the local network structure during invasion can be captured via imaging DISC-3D samples.

### Mass spectrometry imaging of DISC-3D samples

**Figure 5:**
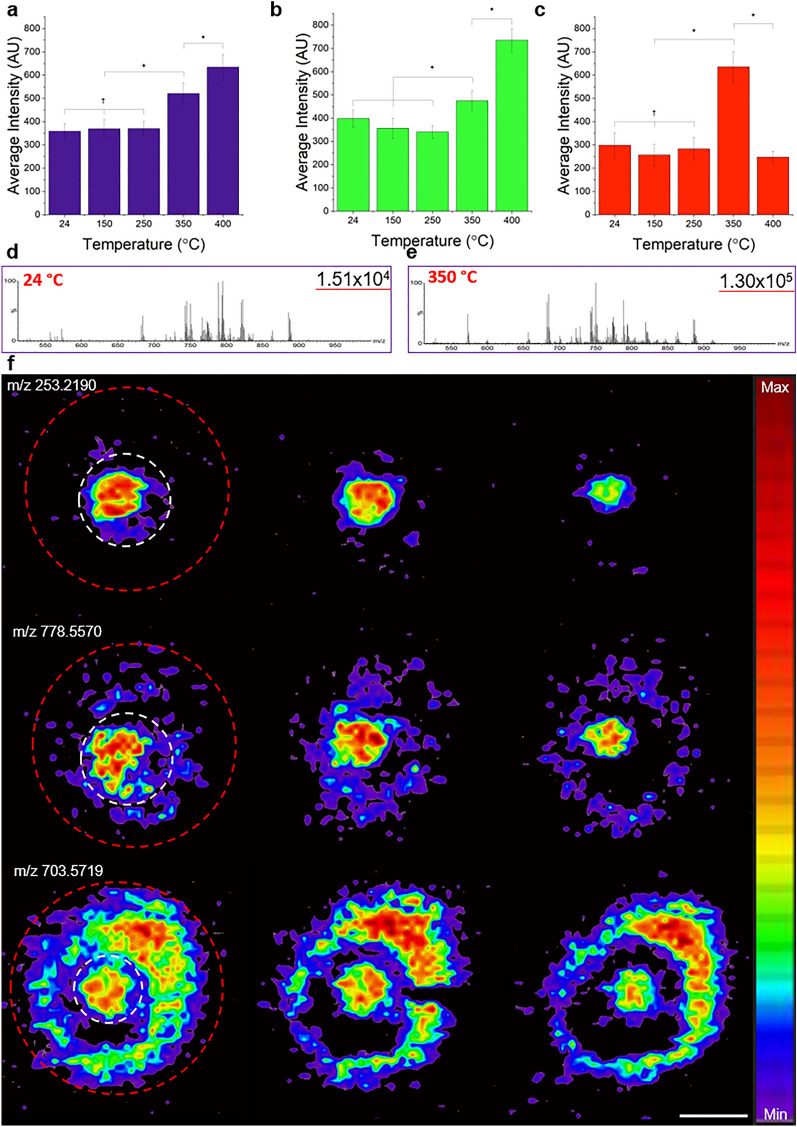
Mass spectrometry imaging of DISC-3D samples (10 μm slices) of MDA-MB-231 spheroids. Average intensity from mass spectrometry signals averaged over spheroid sections at various temperatures across (**a**) the full 200–1000 *m/z* range, (**b**) the 300–500 *m/z* range, and (**c**) the 700–900 *m/z* range. Statistical results shown here were conducted as pairwise samples. A full description of statistical results is presented in Fig. S6b. n = 8 in all cases except 150 °C, where n = 7. *p < 0.05, ^†^p > 0.05. (**d,e**) Representative normalized mass spectra collected from invasive spheroids following the DISC-3D protocol at (**d**) 24 °C and (**e**) 350 °C demonstrating the increase in signal intensity in the ≈ 650–900 *m/z* regime. Number at right on each panel indicates maximum intensity peak at that temperature. (**f**) Representative mass spectrometry images prepared following the DISC-3D protocol outlined in Fig. S1d, showing consecutive sections of single spheroids for several signal ions. In the first serial section of each signal ion, the spheroid core is marked by a white dotted line and the invasive front is denoted by a red dotted line. Color scale from minimum to maximum intensity is shown for signal ions at right. Scale bar = 500 μm.

Having shown the preservation of network structure and optical accessibility of DISC-3D samples, we now assess their suitability for another powerful analytical method, mass spectrometry imaging (MSI). While MSI of spheroid cores has been successfully demonstrated, to date, no such characterization of the invasive front of a spheroid has been achieved^20^. A critical concern with obtaining MSI of DISC-3D prepared samples is the low cellular density at the spheroid invasive front, which leads in turn to a low concentration of molecules of interest in that region. To overcome this challenge, a high-performance modified prototype sprayer and a novel heated transfer capillary were employed (Fig. S6a), which allowed collection of MSI data with the transfer capillary set to temperatures from room temperature to 400 °C. Figure 5a–c shows the average signal intensity across a spheroid section in the 200–1000 *m/z* range (Fig. 5a) and two subsets of this range, the 300—500 *m/z* region (Fig. 5b) and the 700—900 *m/z* region (Fig. 5c). As temperature increases to 350 °C, there is a significant gain in overall signal, a trend that continues as the temperature increases to 400 °C. However, between 350 and 400 °C there is also a significant increase in signal in the 300—500 *m/z* region. This, together with the fact that signal intensity decreases at 400 °C in the 700–900 *m/z* range, suggests fragmentation of lipids and proteins. In contrast, signal is maximized at 350 °C in the 700–900 *m/z* region, where several lipid subclasses—including most triacylglycerols and phosphatidylcholines—have signature signals^49,50^. This suggests 350 °C is the ideal temperature for collecting MSI data on spheroid slices prepared via the DISC-3D protocol, and we detected several lipid classes including free fatty acids, phospholipids, sphingolipids, and glycerolipids in these sections at this temperature. This is further supported by two specific phosphatidylglycerol (PG) lipid ions detected at *m/z* = 773.5338 (PG 36:2) and *m/z* = 747.5182 (PG 34:1), which have been reported to be altered significantly in breast cancers^51,52^. These signals are not only of higher intensity but also their presence throughout the invasive front is most apparent at 350 °C relative to any other temperature (Fig. S6c,d). Exemplary spectra focusing on the 700–900 *m/z* regime collected at 24 °C and 350 °C are shown in Fig. 5d, e, and exemplary spectra of the full *m/z* range at each of the investigated temperatures are shown in Fig. S6e.

Using the prototype heated capillary tube coupled with the DISC-3D protocol, we obtained what are to the best of our knowledge the first mass spectrometry images of lipid distribution in breast cancer cells invading in vitro (Fig. 5f). Interestingly, these images reveal spatial distributions of some lipid ions localized only in the spheroid core, while other lipid ions are present in both the core and the invasive region. For example, we observed that free fatty acids such as FA 16:1 (*m/z* = 253.2190) show preferential localization to the spheroid core while phospholipids such as phosphatidylcholines and ether-linked phosphatidylethanolamines (*m/z* = 778.5570; PE O-40:5) are present throughout both the core and the invasive front. It is particularly interesting that some lipid ions apparent throughout the spheroid are most intense in the invasive region despite the fact that cell density is much lower in that region than in the core, potentially revealing novel lipidomic signatures of invasive cells. Here, we identified sphingomyelin ions [SM 34:1; O2 (*m/z* = 703.5719), Fig. 5f, bottom row and also SM 40:1; O2 (*m/z* = 787.6714)] that show such distribution, consistent with other findings highlighting the increase in high molecular weight sphingomyelins in invasive cancers relative to pre-cancerous breast cancer cells cultured in 3D^53^.

**Figure 6:**
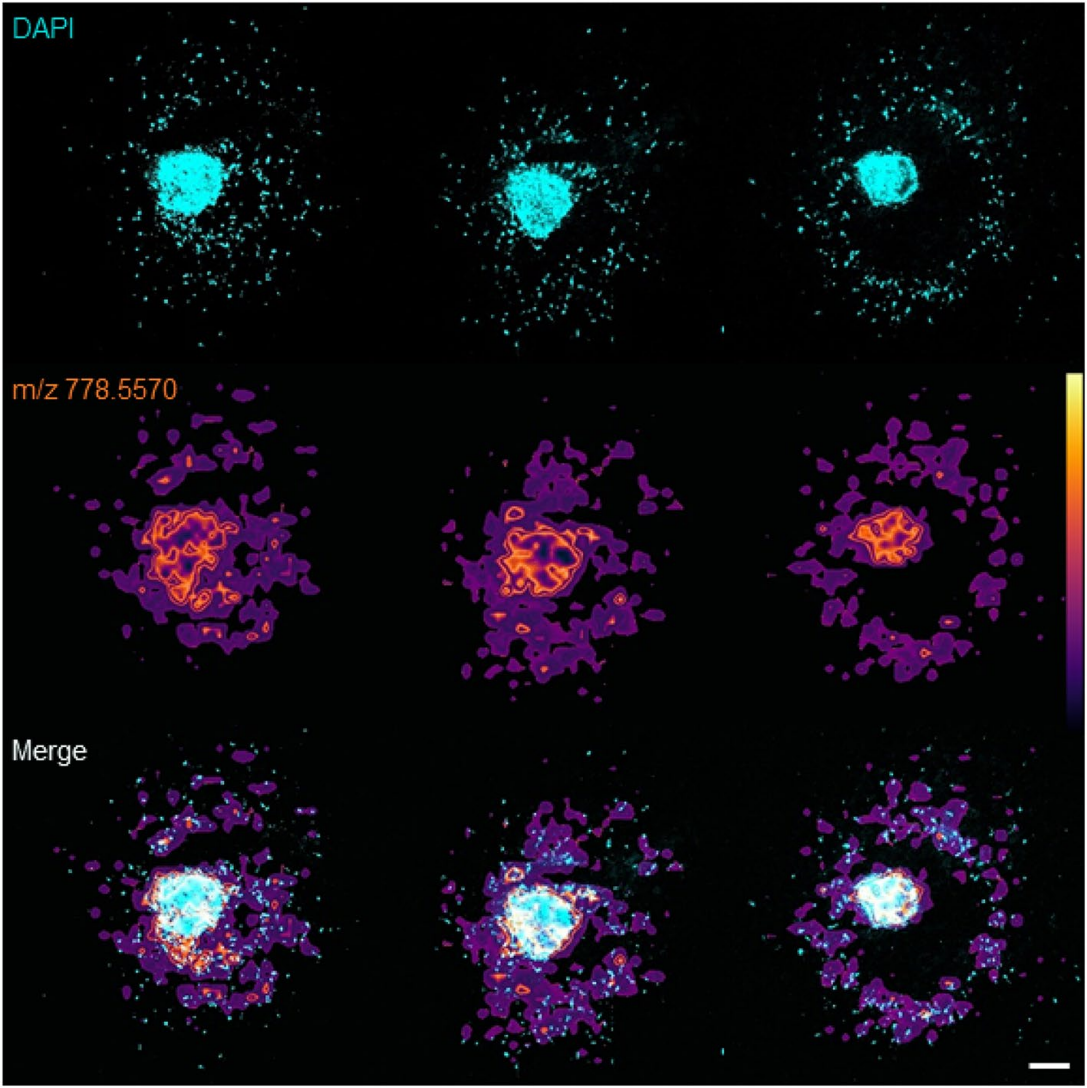
Correlative imaging of consecutive slices of an invasive spheroid prepared using the DISC-3D protocol outlined in Fig. S1d showing (top row) DAPI imaged via confocal fluorescence microscopy, (middle row) MSI signal at 778.5770 *m/z* with linear color scale shown in the right panel, and (bottom row) a correlative image of the confocal and MS images. Scale bar = 200 μm.

### Correlative optical and mass spectrometry imaging of DISC-3D samples

In addition to allowing for a wide-variety of optical microscopies and mass spectrometry imaging, DISC-3D sample preparation facilitates correlative microscopy across these techniques. Such cross-instrument correlative microscopy is typically extremely challenging in 3D samples given their size and dimensionality, which results in a very large search field for identified regions of interest. While fiducial markers can mitigate these challenges, few fiducial markers are appropriate for both MSI and optical imaging and none have been assessed in the context of invasive spheroids^54,55^. Here, we show that DISC-3D facilitates correlative microscopy across instruments without the need for fiducial markers by effectively reducing the search space from 3D to 2D. Figure 6 and Fig. S7 show a variety of correlative images acquired across different instruments and techniques. Of note, registration is successfully performed without fiducial markers even in a hydrogel-only sample that has few distinctive features (Fig. S7).

Figure 6 highlights that correlative imaging of DISC-3D samples can be performed across optical and mass spectrometric imaging techniques, with this figure showing a confocal image of an invading spheroid with cells labeled with the nuclear dye DAPI and an MSI image of this spheroid. Such correlative images assist in interpretation of MSI data, as the optical images clearly reveal cellular density, distributions, and invasive zones. Indeed, this approach could potentially be used to normalize MSI signal as a function of cell density, as normalization remains a challenging problem in MSI^56–58^. We note that labeling cells with DAPI requires cell fixation and permeabilization, which are incompatible with MSI imaging, as the required washing steps remove many lipids of interest. As such, this sample was prepared following the DISC-3D protocol shown in Fig. S1d, mass spectrometry imaging was performed, and then the sample was fixed, permeabilized, and stained with DAPI to enable the optical imaging. This procedure highlights that DISC-3D samples remain appropriate for additional characterization after MSI, which is generally considered a destructive technique.

## Discussion

The DISC-3D protocol allows for interrogation of invasive spheroid samples at high resolution, with high content approaches. In this protocol, typical spheroid culture and invasion is followed by addition of a gelatin solution that is taken through the sol–gel transition before flash-freezing and cryosectioning. The dual collagen and gelatin hydrogel is bi-functional in nature, supporting both cellular invasion and cryosectioning. The sectioning procedure preserves the migration pattern of invading cells as well as the architecture of the collagen matrix, allowing for analysis of spatially heterogeneous cellular behaviors and local alterations of extracellular matrix microstructure via a variety of techniques. Additionally, the DISC-3D protocol does not require fixation or addition of components that may alter sample structure, molecular distribution and molecular detection. This streamlines labeling strategies and allows for application of super-resolution optical imaging and mass spectrometry imaging, techniques not generally possible in 3D cell culture or with established sectioning procedures compatible with invasive spheroids.

Specifically, the DISC-3D protocol facilitates sectioning of 3D collagen I-based hydrogels containing invasive spheroids, avoiding challenges associated with labeling and imaging in the 3D context that can lead not only to poor quality images but also to incorrect understanding of molecular localization in a sample. For example, we showed that while 3D confocal imaging suggests an accumulation of β1 integrin at the spheroid surface (Fig. 2c, top row), imaging these samples after DISC-3D preparation shows that this apparent accumulation of integrin was an artifact caused by poor diffusion of the antibody and high levels of refraction in the intact 3D samples. Additionally, the DISC-3D protocol allows for extended sample storage in advance of staining, facilitating sample re-interrogation when new hypotheses emerge.

We also show that the DISC-3D protocol preserves the collagen I hydrogel fibrillar, microporous structure, a prerequisite for using imaging-based approaches to interrogate cell-extracellular matrix interactions such as occur during cell invasion. Indeed, the DISC-3D protocol results in improved image quality of collagen matrix features across an array of optical microscopy techniques including super-resolution techniques. This is especially obvious deep within the gel, where the in vitro 3D system is most physiologically relevant.

While the improvements that DISC-3D brings to optical imaging over imaging in native 3D samples are striking, the DISC-3D protocol brings additional advantages relative to other sectioning techniques, with sample morphology well preserved compared to previously established sectioning techniques, which is especially important given the highly porous nature of collagen hydrogels. Critically, the approach does not require tissue fixation or the use of OCT, both of which contribute to altered and suppressed lipid signals in mass spectrometry. This allows the DISC-3D approach to be used for mass spectrometry imaging. While MSI is an emerging, powerful technique for characterizing small molecules, lipids, and proteins in biological samples, to this point it has largely been confined to interrogation of tissue slices. While previous researchers have demonstrated MSI of spheroid cores, no existing technique allowed for preservation of invasive cell morphology and provided the high mass spectrometry sensitivity required to characterize the invasive front of spheroids. We demonstrate that the bi-functional, dual collagen I-gelatin hydrogel that is critical in the DISC-3D protocol both supports cellular invasion in 3D and enables cryosectioning while preserving the spheroid core, invasive cells, and surrounding matrix integrity, morphology, and biochemical context. In turn, together with modifications to MSI instrumentation to enhance signal, we demonstrate imaging across the spheroid and local measurement of lipid content in invasive cells. The DISC-3D protocol thus opens the door to lipidomics and metabolomics-based imaging of invasive cells in particular, potentially yielding new insights into the causes of and potential treatments for cancer cell invasion and metastasis.

## Materials and methods

### Reagents

HyClone DMEM high glucose L-glutamine media and porcine gelatin type A 300 bloom strength were purchased from Fisher Scientific (Waltham, MA). Fetal bovine serum and MEM nonessential amino acid solution was obtained from Gibco (Waltham, MA). Accutase and penicillin–streptomycin-amphotericin B, 100X were purchased from MP Biomedicals (Solon, OH). Pepsin-treated (PT) bovine type I collagen was obtained from Advanced BioMatrix (San Diego, CA) as a 5.9–6.1 mg/ml solution. Acid-solubilized (AS) rat tail type I collagen and Cell Recovery Solution were obtained as 10 mg/ml solution from Corning (Corning, NY). ATTO 647N dye (with *N*-hydroxysuccimide (NHS) ester functionality; λ_ex_ = 646 nm, λ_em_ = 664 nm), dimethyl sulfoxide (DMSO), DAPI, 8 M urea, acetic acid (99.7%), and poly-l-lysine solution were purchased from Sigma Aldrich (St Louis, MO). Growth factor-reduced, phenol red-free BME/Matrigel was obtained as an 8.9–12 mg/ml solution from BD Biosciences (San Jose, CA). DMEM solution (10×), NaOH (1 N) and sodium bicarbonate solution (7.5%) were purchased from Sigma Aldrich and sterile filtered before use. Gibco 4-(2-hydroxyethyl)-1-piperazineethanesulfonic acid (HEPES) buffer (1 M), TetraSpeck microspheres (0.1 µm, fluorescent blue/green/orange/dark red) and FluoroSpheres (carboxylate modified, 1 µm Nile Red fluorescent 2% solid) were obtained from Invitrogen (Carlsbad, CA) and sterile filtered before use. Buffered formalin phosphate (10%), bovine serum albumin (BSA), water (LC–MS grade), methanol (LC–MS grade), and formic acid (99%) were obtained from Thermo Fisher Scientific (Pittsburgh, PA). Triton-X was obtained from EMD Millipore Chemicals (Billerica, MA). AlexaFluor488-conjugated anti-integrin β1 antibodies (clone P5D2, staining total β1), and AlexaFluor647-conjugated goat anti-mouse secondary antibody were obtained from Abcam (Cambridge, MA). AlexaFluor 555 NHS ester and AlexaFluor-conjugated phalloidin were obtained from Molecular Probes by Thermo Fisher Scientific (Waltham, MA). MT1-MMP AlexaFluor488 conjugated antibody was obtained from R&D Systems (Minneapolis, MN). Anti-p53 antibody and anti-RhoA antibody were a gift from Carol Prives’ Lab (Columbia University, NY). 1 M tris HCl buffer was purchased from Lonza Accugene (Morristown, NJ). Vectashield mounting media was purchased through Vector Laboratories (Burlingame, CA).

### Materials

Pre-cleaned microscope slides 24 × 75 × 1 mm, coverslip glass 24 × 40, and Nunclon Sphera plates were purchased from Fisher Scientific (Waltham, MA). High tolerance dishes (P35G-0.170-14-C) were obtained from MatTek Corporation (Ashland, MA). 35 mm fluorodishes were obtained from World Precision Instruments (Sarasota, Fl). Silicone culture inserts for self-insertion were purchased from Idibi (Grafelfing, Germany).

### Cell lines and cell culture

MDA-MB-231 breast cancer cells were obtained from the American Type Culture Collection (Manassas, VA). Cells were cultured in 1× high glucose DMEM containing 10% (v/v) fetal bovine serum, 1% (v/v) 100× antibiotics solution, and 1% (v/v) 100× non-essential amino acids solution. Cells were maintained at 37 °C under 5% carbon dioxide. Cells were subcultured when 80–90% confluency was reached and were used from passages 3–20.

### Generation of multicellular tumor spheroids

Spheroids were generated using a modification of the protocol described previously.^32^ Specifically, cells plated on tissue culture plastic were detached using Accutase and placed in ice cold DMEM growth culture medium containing 0.2575 mg/mL Matrigel (basement membrane extract), 1 × 10^–4^% (v/v) Nile red fluorospheres (as appropriate), and 5 × 10^4^ cells/mL, resulting in spheroids containing 10,000 cells each. For spheroids containing fluorospheres, there was an initial centrifugation containing half of the growth culture medium and the fluorospheres. Following this, the remaining growth culture medium containing the cells and Matrigel was carefully pipetted on top of the growth cell culture medium containing the fluorospheres, and the plate was centrifuged again. In the absence of fluorospheres, only a single centrifugation containing all components mentioned above occurs. Centrifugation was conducted in ultralow attachment 96-well U-bottom Nunclon Sphera plates at 4 °C, 1000G, for 10 min. Spheroids were allowed to compact at 37 °C under 5% carbon dioxide for 48 h.

### Fluorescent labeling of collagen I

Fluorescently labeled collagen I was generated as described previously.^59,60^ In brief, AS rat tail collagen I was diluted to a 2 mg/mL solution in sterile filtered 20 mM acetic acid and 0.01 M sodium bicarbonate to a pH of ≈ 8. NHS ester-modified AlexaFluor555 was added to the collagen I solution to achieve the desired labeling ratio (generally 5 fluorophores per collagen I monomer), and the reaction was allowed to proceed at 4 °C in the dark for 24 h. The labeled collagen solution was then dialyzed against 20 mM acetic acid for 3 days to remove any unbound dye. Labeled collagen I was stored in the dark at 4 °C for up to 6 months.

### Generation of cell free collagen gels

Collagen gels were prepared from bovine type I high-concentration (5.9–6.1 mg/mL) pepsin treated collagen monomer stock solution. Solutions were generated from 10% (v/v) 10× DMEM, 2.5% (v/v) HEPES buffer, 2.5% (v/v) sodium bicarbonate, and cell culture grade water. This procedure was done entirely on ice at 4 °C to prevent collagen self-assembly. Collagen was added to the solution such that a final collagen I concentration of 1 mg/mL was obtained, and the solution was adjusted to a pH of 7.4 using NaOH. Solutions were allowed to gel at 37 °C and 5% carbon dioxide for 45 min to 1 h and were subsequently overlaid with cell culture growth medium to prevent dehydration of the gel. Gels with fluorescently labeled collagen I were generated in the same manner, with the exception being that fluorescently labeled collagen monomers were added in a 1:10 v/v ratio to unlabeled collagen.

Once prepared, solutions were pipetted into custom made chambers. These chambers were constructed from cell culture inserts with the center removed. Cell culture inserts were then attached to coverslip-bottom cell culture dishes via the adhesive present on the inserts. 150 μL of solution was pipetted into each chamber, and the chambers were quickly transferred to 37 °C and 5% carbon dioxide for 45 min to 1 h to allow for gelation. Gels were subsequently overlaid with 100 μL cell culture growth medium, and ≈ 600 μL of growth medium was added to the area surrounding the gels to prevent dehydration of the gel.

### Generation of collagen gels containing spheroids

Collagen gels with implanted spheroids were prepared as described above, with the addition of individual spheroids to the solution before gelation. Prior to spheroid addition to the solution, spheroids were placed in ice-cold Cell Recovery Solution for 45–60 min to remove any Matrigel from the exterior of the spheroid that accumulated during spheroid formation and compaction. During this process, collagen solutions were made and poured as described above, such that following treatment with Cell Recovery Solution a single spheroid could be placed into each individual collagen solution. Solutions were then incubated and overlaid with growth culture medium as described above.

### DISC-3D protocol part 1—preparing spheroids and gels for cryosectioning

As is schematically depicted in Fig. 1 and Fig. S1a, the DISC-3D protocol begins with forming spheroids by centrifuging cells in the presence of basement membrane extract (Matrigel), as described above. The spheroid solution is supplemented with Nile Red fluorospheres (polystyrene, 1 μm diameter) prior to centrifugation, which act as a visual cue for the location of the spheroid during cryosectioning. The fluorospheres do not impact the invasive capacity of the cells (Fig. S2). Following centrifugation, spheroids are allowed to compact for 48 h and are then implanted into 1 mg/mL collagen I solutions (Fig. S1a), and transferred to an incubator at 37 °C and 5% CO_2_ to initiate collagen gelation and maintain cell health.

From this point, the DISC-3D protocol has three parallel sample preparation pathways appropriate for distinct experiments (Fig. S1b–d). In all three approaches, 10% (w/v) porcine type A gelatin is used to form a secondary network. All gelatin used in this work is autoclaved prior to use. This decreases the average gelatin molecular weight and gelatin solution viscosity, allowing the solution to permeate the collagen network more readily. Following autoclaving, gelatin solutions were stored at 37 °C prior to being added to the samples.

#### DISC-3D protocol for spheroid optical imaging (Fig. S1b,c)

To prepare samples for imaging of the spheroid core, samples were allowed to gel at 37 °C and 5% CO_2_ for 1 h, at which point they were fixed and stained (Fig. S1b). To prepare samples for imaging the spheroid core and invading cells, spheroids were allowed to invade for 48 h following collagen I implantation. At this point, samples were fixed and stained (Fig. S1c). In both cases, following staining (described in the “Immunocytochemistry” section below), the samples were overlaid with 75 μL of PBS and 100 μL of 10% gelatin. Approximately 600 μL of PBS was added to the area surrounding the gels to prevent dehydration of the gel. Once the sample had been overlaid with gelatin, the sample was incubated at 37 °C for 24 h. The sample was then held at 4 °C for 1 h to allow for gelatin gelation, after which the sample was flash frozen in liquid nitrogen and stored at −80 °C until it was cryosectioned.

#### DISC-3D protocol for MSI (Fig. S1d)

Invasive spheroids prepared for mass spectrometry imaging were allowed to invade for 24 h. At that point, spheroids were overlaid with 10% (w/v) porcine gelatin type A. The samples were then incubated at 37 °C for another 24 h, allowing the cells to continue migrating unimpeded as the gelatin diffused through the sample (Fig. S2). Following this incubation period, the samples were chilled at 4 °C for 1 h to allow for gelatin gelation. The sample was then flash frozen in liquid nitrogen and stored at −80 °C until it was cryosectioned.

#### DISC-3D protocol for cell free gels

Once cell-free collagen gels were completely gelled at 37 °C (1 h), they were overlaid with 75 μL PBS and 100 μL of 10% gelatin. The sample was then incubated at 37 °C for 24 h, gelled at 4 °C for 1 h, flash frozen, and stored at −80 °C until cryosectioning.

Samples prepared to this stage are generally viable for up to 1 month when stored in an air tight container at −80 °C and may be used for as long as 6 months, but longer time scales result in drier samples and therefore increased sample cracking during cryosectioning.

### DISC-3D protocol part 2—cryosectioning 3D samples

Samples were cryosectioned in a Leica CM3050 S cryostat with both the chamber and sample holder held at −20 °C. Temperatures below −20 °C tended to result in sample cracking, and temperatures above −20 °C increased sample likelihood of adhering to the stage, which inhibits clean sectioning. Samples were allowed to warm to −20 °C for at least 20 min prior to cutting to prevent fracturing. The silicone cell culture spacers containing the gelled samples were detached from the cell culture dish by carefully tipping the sample off the dish, as pulling the samples off the dish often resulted in sample cracking. Samples were then removed from the silicone spacers and mounted to the chuck using mass spectrometry grade water.

Samples were mounted such that the orientation of the gel on the chuck mimicked the orientation of the gel on the cell culture dish (i.e., the bottom of the gel remains the bottom in both scenarios). Samples were trimmed to the region of interest (using the visual cue of the Nile Red fluorospheres when applicable). Samples were then sliced to the desired thickness: 4 μm for collagen only gels and 10 μm for collagen gels containing spheroids. Collagen I slices had a tendency to adhere to surfaces and crumple, making use of the built-in roll-guard on the cryostat challenging. As such, sample rolling was prevented by carefully guiding cut slices off the sample using a soft tipped paintbrush. Samples were then transferred to the appropriate glass slides, again using a soft tipped paint brush (Pre-cleaned Microscope Slides 24 × 75 × 1 mm for mass spectrometry imaging, Coverslip Glass 24 × 40 × 0.13–0.17 for microscopy imaging, and 35 mm high tolerance dishes P35G-1.0-14-C for super resolution microscopy). Following cryosectioning, samples can be stored in an airtight container at −80 °C until ready for use. To date, no sample degradation in the −80 °C freezer has been seen, including for samples stored up to 1 year. Once ready for use, samples should be dried in a desiccant under vacuum for at least 10 min. Following drying, samples can be further stained and labeled, as described below.

### Immunocytochemistry and labeling for specific experiments

#### Immunocytochemistry and labeling of fixed (non-invasive) spheroids

Following spheroid formation and implantation, spheroids were fixed at the 1-h time point with pre-warmed 4% formalin for 20 min. Spheroids were then rinsed three times for 10 min with 1× PBS. Next, spheroids were permeabilized for 10 min in 0.02% Triton-X solution, followed by a quick rinse, two 10-min soaks, and one 30-min soak, all in 1× PBS. Spheroids were then stained and imaged as described below.

#### Anti-β1 integrin (clone P5D2)/DAPI/phalloidin (DISC-3D protocol in Fig. S1b; Fig. 2)

Anti-β1 integrin AlexaFluor488 (clone P5D2) was stained at a concentration of 1:200 (v/v), phalloidin AlexaFluor 647 was stained at a concentration of 1:1000 (v/v), and DAPI at a concentration of 1:1000 (v/v). Samples were stained overnight in the dark at 4 °C and were subsequently rinsed three times for 10 min with 1× PBS. Samples were overlaid with 100 μL of 1× PBS and then imaged (while still in 3D) on the Zeiss LSM800 using a 20× objective. Z-stacks were taken at 10 μm intervals as high as the working distance of the objective would allow. Samples were then overlaid with gelatin and PBS, frozen and cryosectioned (DISC-3D Protocol Part 2). Every slice (typically, ≈ 50) of the spheroid was imaged on the Zeiss LSM800 using the 20× objective. Each of these slices was then re-stained using the same dyes and concentrations listed above. Approximately 20 μL of the staining solution was placed atop each individual slice of the cryosectioned spheroid, and the samples were allowed to sit overnight at 4 °C in a humidified and sealed chamber (so as to reduce evaporation of the staining solution). The samples were rinsed the next morning three times in 1× PBS for 10 min, dried completely, and every slice was again imaged as described above.

#### Anti-β1 integrin (clone P5D2)/DAPI/phalloidin/fluorospheres (DISC-3D protocol in Fig. S1b; Fig. S3)

Spheroids were stained with anti-β1 integrin AlexaFluor488 (clone P5D2) at a concentration of 1:200 (v/v) and DAPI at a concentration of 1:1000 (v/v). Samples were stained overnight in the dark at 4 °C and were subsequently rinsed three times for 10 min with 1× PBS. Samples were overlaid with gelatin and cryosectioned as described in DISC-3D Protocol Part 2. Images of cryosectioned samples were obtained on the Zeiss LSM800 using the 20× objective. Samples were then stored in an airtight, desiccated box at 4 °C for 10 months. The sample was warmed to room temperature and was then stained with phalloidin AlexaFluor 647 at a concentration of 1:1000 (v/v). The stain was added at 20 μL per cryosectioned slice and was allowed to sit in a sealed, humidified chamber at 4 °C overnight. The sample was subsequently washed three times in 1× PBS for 5 min. Following sectioning, all samples were imaged on the Zeiss LSM800 Airyscan on the 63× oil objective.

#### Immunocytochemistry and labeling of fixed invasive spheroids

Samples of invasive spheroids (all prepared following the DISC-3D Protocol outlined in Fig. S1c) were generated as described previously and were fixed after 48 h of invasion. Cell culture growth medium was removed from the gels. The gels were then fixed for 20 min using pre-warmed 4% methanol-free formaldehyde in 1× PBS for 20 min, followed by three 10-min rinses with PBS. Samples were then permeabilized using 0.02% Triton-X in 1× PBS for 10 min. The Triton-X was then removed, and the samples were quickly rinsed with PBS and were then washed twice for 10 min in 1× PBS, followed by a 30 min soak in 1× PBS. Samples were then stained as described below in 100 μL of 1× PBS per chamber and overnight at 4 °C unless otherwise stated. Following staining, samples were rinsed three times with 1× PBS for 10 min and overlaid with 75 μL of 1× PBS. Samples were then overlaid with gelatin and cryosectioned following the procedures outlined above (DISC-3D Protocol Part 2). Following sectioning, all samples were imaged on the Zeiss LSM800 in Airyscan mode using the 63× oil objective.

#### Anti-RhoA and DAPI (Fig. 3b,f)

The directly-conjugated anti-RhoA antibody was used at a concentration of 1:100 (v/v) and DAPI at a concentration of 1:1000 (v/v). Following these stainings, the samples were washed three times for 10 min in 1× PBS and blocked for 1 h at room temperature in a 2% BSA solution. The samples were subsequently stained with a secondary antibody using a mouse monoclonal IgG fluorescently labeled with ATTO 647 at a concentration of 1:100 v/v in a 2% BSA solution at room temperature for an hour. Samples were then treated as described in “Immunocytochemistry and labeling of fixed invasive spheroids”.

#### Anti-p53 and DAPI (Fig. 3c,g)

The directly-conjugated anti-p53 antibody was used at a concentration of 1:1 (v/v), with DAPI added at a concentration of 1:1000 (v/v). The samples were then washed three times for 10 min in 1× PBS and were blocked for 1 h at room temperature in a 2% BSA solution. The samples were subsequently stained with a secondary antibody using a mouse monoclonal IgG fluorescently labeled with ATTO 647 at a concentration of 1:100 v/v in a 2% BSA solution at room temperature for an hour. Samples were then treated as described in “Immunocytochemistry and labeling of fixed invasive spheroids”.

#### Anti-MT1-MMP and DAPI (Fig. 3d,h)

MT-MMP-1 AlexaFluor 488 antibody was applied at a concentration of 1:10 (v/v) and DAPI was stained at a concentration of 1:1000 (v/v) Samples were then treated as described in “Immunocytochemistry and labeling of fixed invasive spheroids”.

### Optical imaging

Optical microscopy was performed on one of four microscopes. An Olympus Fluoview 300 inverted confocal laser scanning microscope with a 10× air (NA 0.4) objective was used for Fig. S2. A Zeiss LSM700 inverted confocal laser scanning microscope with a 63× oil (NA 1.4) objective was used for images labeled “confocal” in Fig. 4a and Fig. S7. A Zeiss LSM800 confocal laser scanning microscope 63× oil (NA 1.4) was used for Figs. 2a–c, 6, and Fig. S3. A Zeiss LSM800 confocal laser scanning microscope with a 10× (NA 0.4) objective was used for Fig. 3a, e. That same microscope in Airyscan mode with a 63× oil (NA 1.4) was used for Figs. 3b–d, f–h, 4a, S5 and S7 for images labeled “Airyscan.” A Zeiss Elyra 7 microscope 63× oil (NA 1.46) was used in either widefield mode, lattice SIM mode, or STORM mode as needed for Fig. 4a and Fig. S7.

### Super-resolution image acquisition and reconstruction

Airyscan images were obtained on a Zeiss LSM800 inverted confocal microscope with a 63× oil (NA1.4) objective equipped with a GaAsP-PMT detector. Optimal settings for pixel size, scan speed, and scan area were used as defined by the ZeissZen Blue software. Following image collection, images were processed using the Airyscan processing tool in the ZeissZen Blue software.

SIM images were obtained on a Zeiss Elyra 7 microscope equipped with lattice SIM using a 63× oil (NA 1.46) objective, an additional 1.6× magnification lens, and pco.edge 4.2 sCMOS camera. Optimal settings for grating and z stack resolution were used as defined by the ZeissZen Black software. SIM images were reconstructed using the default settings in the ZeissZen Black software.

For STORM imaging, imaging buffer was prepared as described previously.^61^ In brief, 1 M pH  8.0 Tris buffer was prepared. Vectashield mounting media H1000 was diluted in 95% (v/v) glycerol to yield a final concentration of 25% (v/v) mounting media. Tris buffer was added to yield a final concentration of 50 mM Tris in the complete imaging buffer. STORM images were acquired on a Zeiss Elyra 7 equipped with a 63× 1.46 NA TIRF objective and pco.edge 4.2 sCMOS camera. Images were collected using HILO illumination. Samples were illuminated with 100% laser power to induce dye blinking with a 561 nm laser (100 mW). Images were acquired over 30,000 frames with a 20 ms exposure time. STORM images were reconstructed using the ThunderSTORM plugin in FIJI with default analysis settings. Camera settings for reconstruction were set according to manufacturer specifications [pixel size = 97 nm, photoelectrons per A/D count = 0.46, base level (A/D counts) = 97]. Localized molecules in reconstructed images were filtered by intensity (> 100 photons) and standard deviation of the fitted Gaussian (> 50 nm and < 250 nm) to remove noise and poor-quality fits. Noise was further reduced by using a DBSCAN-based filter to remove outlier molecules in sparsely populated regions of the imaging area (Epsilon = 50 nm, MinPts = 5). After these post-processing steps, images were drift-corrected using cross-correlation with a bin size of 5. Molecules in post-processed and drift-corrected images were visualized using the “Normalized Gaussian” rendering with a lateral uncertainty set to 10 nm.

### Mass spectrometry imaging

Spheroids prepared for mass spectrometry imaging were formed and implanted into collagen gels as described above, following the DISC-3D Protocol outlined in Fig. S1d. The samples were then cryosectioned into 10 μm thick slices and MSI images were obtained.

The sections were imaged on a Prosolia Desorption Electrospray Ionization (DESI) 2D stage mounted on the SYNAPT HDMS G2-Si Q-ToF mass spectrometer (Waters Corporation, Milford, MA, USA). The DESI was equipped with a Waters high-performance modified prototype sprayer and a prototype heated transfer capillary to optimize transfer of generated ions (Fig. S6). The electrospray solvent consisted of methanol/water/formic acid (98:2:0.01; v/v/v) containing 40 pg/µL of leucine enkephalin as internal lock mass. The flow rate was 2 µL/min. The spray capillary voltage was set to 0.6 kV, the cone voltage was 50 V, and the ion source temperature was set to 150 °C. Mass spectra were acquired using positive and negative ionization modes with the mass range of *m/z* 50–1200 and a pixel size of 40 μm. The heated transfer line was set at temperatures ranging from 24 to 400 °C. Ion image mass spectral data (corresponding *m/z* features in every pixel within the image) from DESI MSI was processed for visualization using High Definition Imaging (HDI version 1.6, Waters Corporation) software. All the images were normalized to the total ion current. The lipid ions were annotated by searching monoisotopic masses against the available online databases Lipid MAPS with a mass tolerance of 5 ppm and matching the drift times with the available standards.

### Correlative imaging

Correlative imaging across optical microscopy techniques was performed on cell free hydrogels taken through the DISC-3D protocol as described above (Fig. S7). Samples were imaged in order on the Zeiss LSM700 (confocal), Zeiss LSM800 in Airyscan mode, the Zeiss Elyra SIM, and finally the Zeiss Elyra STORM. All image correlation was performed using the FIJI plugin TurboReg^62^.

Spheroids prepared for both mass spectrometry imaging and confocal microscopy were generated and implanted as described previously (Fig. S1d) and were cryosectioned as described in the DISC-3D protocol Part 2. Following cryosectioning and DESI MSI acquisition, spheroids were fixed using 4% formalin for 10 min, followed by three 5-min washes using 1× PBS. The sample was then stained with DAPI at a concentration of 1:1000 (v/v) in 1× PBS. Approximately 20 μL of the dye solution was placed atop each cryosectioned slice. Samples were stained overnight in the dark at 4 °C in a sealed, humidified chamber. Subsequently, samples were rinsed three times for 5 min in 1× PBS. Spheroid slices were imaged on the Zeiss LSM800 microscope at 20× magnification. Image correlation was done using the FIJI TurboReg registration plugin.

### Image analysis and statistics

All statistical tests were conducted at an α value of 0.05. Statistical significance was marked using asterisks, where * denotes a p < 0.05, ** denotes a p < 0.01, and *** denotes a p < 0.001. A lack of statistical significance (p > 0.05) is denoted by a dagger (†). A  denotes statistical significance and is described in the relevant figure caption when employed.

#### Spheroid invasion

Spheroid invasive distances (Fig. S2) were determined using 10× images taken on the Olympus Fluoview 300 in scanning transmittance mode at t = 1 h and t = 48 h post implantation of the spheroids. The invasive distance was defined as the circumference of a circle that encompasses at least 90% of the invasive front of the spheroid at t = 48 h, minus the circumference of a circle that fully encompasses the spheroid core at t = 1 h.

#### Signal intensity across spheroids

The fluorescence intensity across the spheroid in 3D, cryosectioned, and cryosectioned and re-stained spheroids (Fig. 2) was calculated in FIJI by drawing a line bisecting the spheroid. The intensity profile along this line was normalized to the maximum intensity along that line and plot. Normalized signal intensity was calculated for every imaged slice that was available—12 slices for the 3D sample, 56 slices for the DISC-3D samples, and 49 slices for DISC-3D re-stained samples. Average intensity for each slice was determined and plot as box and whisker plots (Fig. 2i–k).

#### Signal to background ratio

Signal to background ratios of 3D and cryosectioned images (Fig. 3) were determined by dividing the maximum intensity of regions of interest encompassing cells (or the local extracellular environment in the case of MT1-MMP) to the maximum intensity of regions outside the cells and/or the local extracellular environment.

#### Collagen images

To determine apparent collagen fiber width (Fig. 4b), ROIs were drawn across collagen fibers perpendicular to the longest dimension of the fiber. Pixel intensity values were recorded along the ROI, and the intensity profile was fit to a Gaussian function of the form:

1 $$\mathrm{y}={\mathrm{y}}_{0}+\mathrm{A}*{\mathrm{e}}^{\frac{-(\mathrm{x}-{\mathrm{x}}_{0})}{2{\upsigma }^{2}}}$$

All fits with an adjusted R^2^ value below 0.95 were discarded before performing further analysis. The full width at half maximum (FWHM) of these curves were extracted, and the mean value defined apparent fiber width.

Determination of resolution of widefield, confocal, Airyscan and SIM microscopies was performed by measuring the FWHM of the point spread function of a point source. Specifically, TetraSpeck beads (100 nm) were deposited on glass that was first coated with 0.01% poly-l-lysine for 10 min at room temperature. The poly-l-lysine solution was removed and the glass slide was allowed to dry. Beads were diluted 1:200 in dIH_2_O, sonicated for 5 min, and then vortexed. The bead solution was added to the glass slide and set aside for 10 min. Excess solution was removed and the beads were imaged using each of the respective microscopy methods in configurations mirroring those used for imaging collagen fibers. FWHM of the point spread function of the beads using each of these techniques was determined in the same manner collagen I fiber widths were determined. In brief, ROIs were drawn across the center of the bead, and the intensity across the ROI was fit to a Gaussian curve. The FWHM of the curve defined the resolution and minimum expected fiber width associated with each technique.

Resolution of STORM imaging was determined using:

2 $$Resolution=\sqrt{{\left(Localization \, Precision\right)}^{2}+ {\left(Nyquist \, Resolution\right)}^{2}}$$

as described in Ref.^53^. Localization precision was computed in the FIJI plugin ThunderSTORM, following the protocol outlined in Ref.^63^. Nyquist resolution was calculated using the protocol described in Ref.^64^, which relates image resolution to the localization density of the image. Fig. S4d shows the change in resolution and apparent collagen fiber width (using the method described above) as a function of number of frames included in the analysis. Both quantities approach plateaus by approximately 20,000 frames, supporting the choice of 30,000 frames as the number used for STORM imaging and analysis thereof.

Pore size was determined using the method described in Ref.^65^. To reduce any potential bias, the same image processing workflow was used for all imaging contexts (across microscopy techniques, and 3D high/low vs. DISC-3D). In ImageJ, raw images were subjected to a bandpass filter and rolling ball background subtraction to remove features below a certain size threshold and reduce out of plane background signal, respectively. Images were then subjected to an auto local threshold to obtain a binarized image. To determine pore size, the distances between “on” pixels were counted row by row and column by column in the segmented image. The distribution of these gaps between fibers was fit to the exponential probability density function:

$$f\left(x\right)=\lambda *{e}^{(-\lambda *x)}$$

The pore size reported for the network is the characteristic pore size 1/λ.

## Supplementary Information

Supplementary Figures.

## Data Availability

Data will be made available on request.
