# Supplementary material for: DISC-3D: dual-hydrogel system enhances optical imaging and enables correlative mass spectrometry imaging of invading multicellular tumor spheroids
Source: Sci Rep. 2023 Jul 31;13:12383. doi: 10.1038/s41598-023-38699-1 (PMC10390472; doi:10.1038/s41598-023-38699-1)
Supplement: Supplementary file 1 — Supplementary Figures. [file 41598_2023_38699_MOESM1_ESM.pdf]

Supplementary Information

DISC-3D: dual-hydrogel system enhances optical imaging and enables correlative mass spectrometry imaging of invading multicellular tumor spheroids

Rachel C. Avard[1], Megan L. Broad[1,2], Fereshteh Zandkarimi[1], Alexander J. Devanny[1], Joseph L. Hammer[1], Karen Yu[1,3], Asja Guzman[4], Laura J. Kaufman[1]*

[1] Department of Chemistry, Columbia University New York NY 10027
[2] Department of Chemistry, Cardiff University Cardiff Wales UK CF10 3AT
[3] Department of Physics, Columbia University New York NY 10027
[4] Department of Biological Sciences, Columbia University New York NY 10027

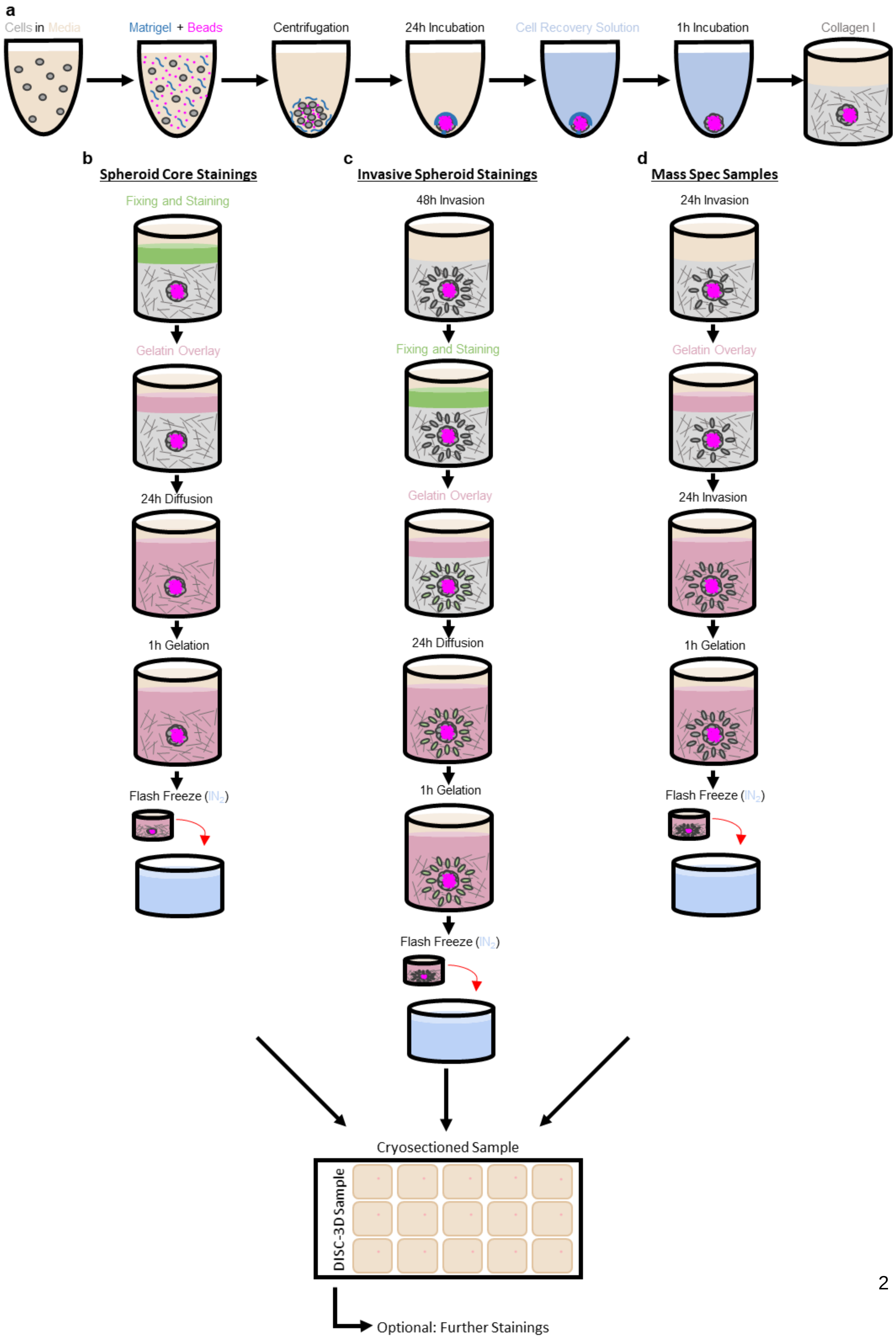

a
Cells in Media
Matrigel + Beads
Centrifugation
24h Incubation
Cell Recovery Solution
1h Incubation
Collagen I
b
Spheroid Core Stainings
Fixing and Staining
Gelatin Overlay
24h Diffusion
1h Gelation
Flash Freeze (lN_2)
c
Invasive Spheroid Stainings
48h Invasion
Fixing and Staining
Gelatin Overlay
24h Diffusion
1h Gelation
Flash Freeze (lN_2)
d
Mass Spec Samples
24h Invasion
Gelatin Overlay
24h Invasion
1h Gelation
Flash Freeze (lN_2)
Cryosectioned Sample
DISC-3D Sample
Optional: Further Stainings

Figure S1. Possible variations of the DISC-3D Protocol. (a) Initial steps of the DISC-3D protocol, in which spheroids are created via centrifugation in the presence of Matrigel (a compaction agent) and Nile Red fluorospheres, which act as a visual cue. (b-d) The 3 main variations of the DISC-3D protocol. (b) Protocol used for spheroid core interrogation, in which spheroids are fixed and stained 1 hour post implantation into collagen. Samples are then overlaid with gelatin, and 24 hours later the gelatin is gelled and the sample is flash frozen. (c) Protocol for interrogation of invasive spheroids, in which spheroids are implanted into collagen I and allowed to invade for 48 hours, at which point the spheroids are fixed, labeled, and overlaid with gelatin. 24 hours later the gelatin is gelled and the sample is flash frozen. (d) Protocol used for MSI, in which spheroids are allowed to invade for 24 hours, at which point they are overlaid with gelatin. The spheroids are allowed to invade for another 24 hours as the gelatin permeates the sample, at which point the gelatin is gelled and the sample is flash frozen. Following flash freezing, all samples were cryosectioned to 10 μm slices. Samples can be further labeled at any point following cryosectioning, including after both optical and mass spectrometry imaging, as well as after long term storage.

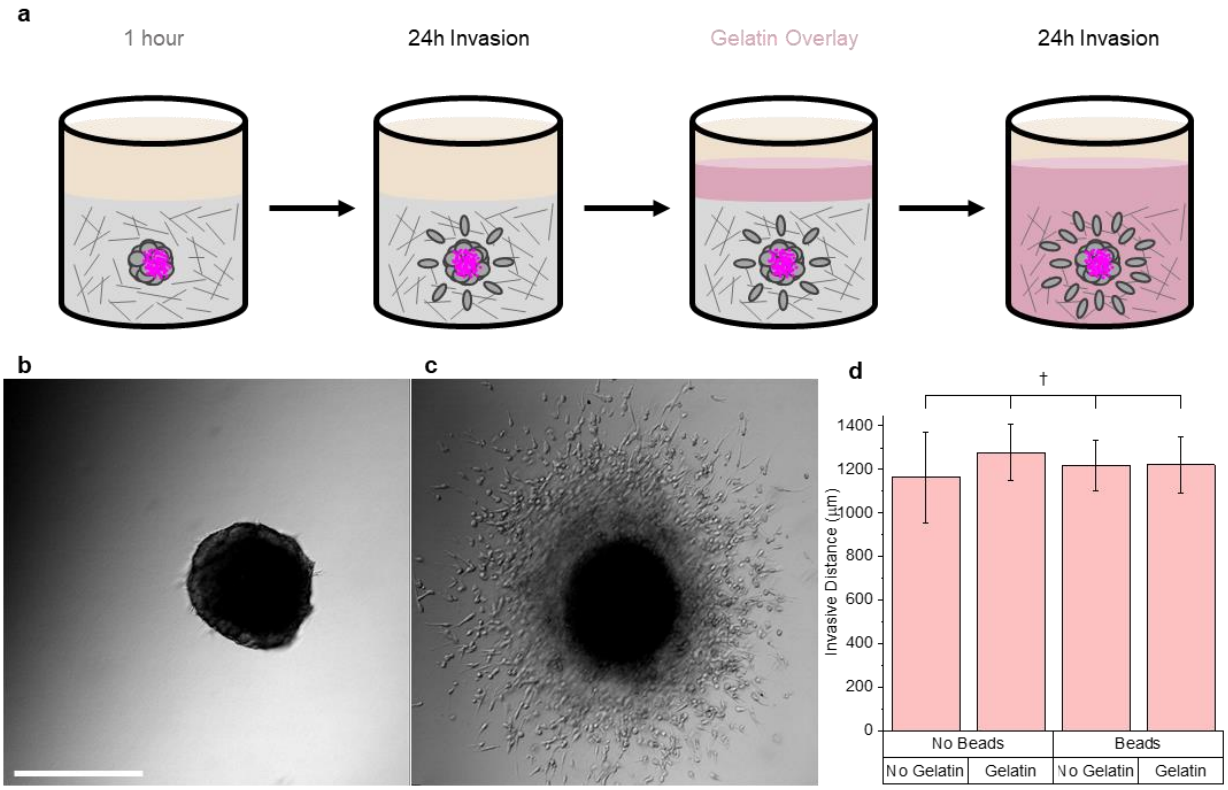

Figure S2. Addition of gelatin and beads does not impact spheroid invasion. (a) Schematic depiction (from Fig. S1d) of a spheroid implanted in a collagen network, allowed to invade for 24 hours, overlaid with gelatin, and allowed to invade for another 24 hours. (b,c) A representative MD-MBA-231 spheroid at two points in the DISC-3D protocol, as outlined in blue in Fig. 1 in the main text and in the first and last image in (a) above; (b) Representative spheroid 1 hour post-implantation and (c) 48 hours post-implantation (and 24 hours post-gelatin addition). Scale bar = 500 μm. (d) Invasive distance for spheroids prepared either typically (no beads, no gelatin) or with one or both additions (fluorescently labeled beads and gelatin solution) used in the DISC-3D protocol as outlined in Figure 1 and Methods in the main text. Invasive distance is defined by the distance the majority (≈ 90%) of the invasive front reaches during the invasive period and is described more fully in the Methods section. Neither the addition of beads nor gelatin nor a combination of the two impact the invasive capacity of the cells. † $p > 0.05$. [n = 9]

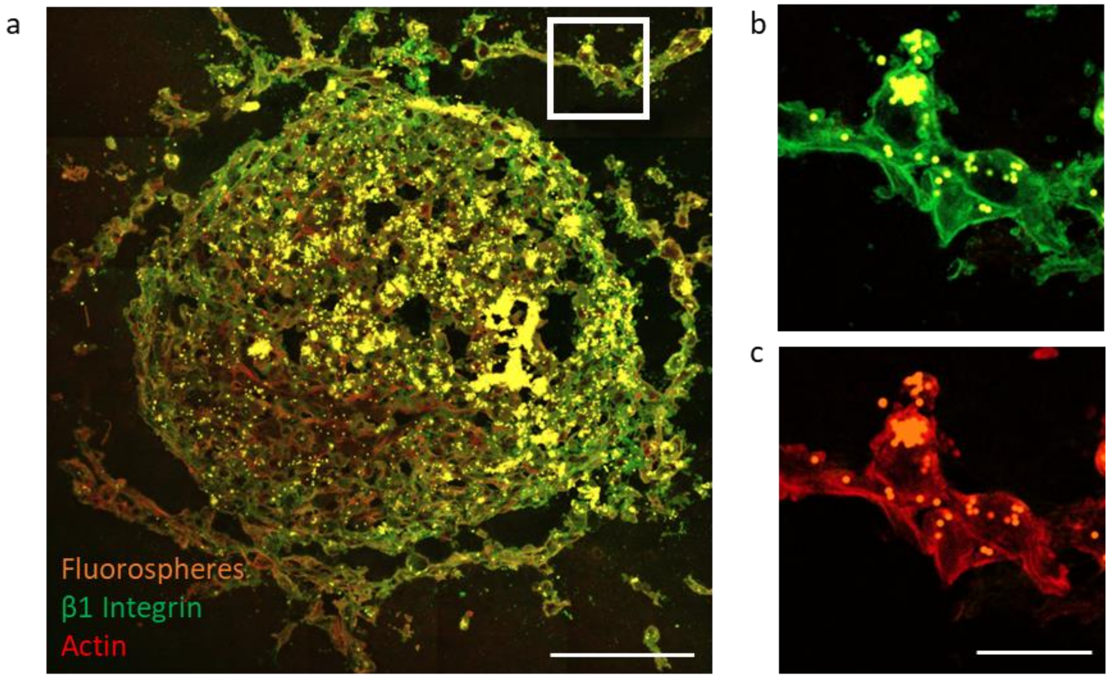

Figure S3. Images of a spheroid prepared using the DISC-3D protocol outlined in Fig. S1c and stored for 10 months. The sample was initially stained for β1 integrin immediately after sample processing, stored for 10 months, and then re-stained with phalloidin (for f-actin) (a) Spheroid slice after storage showing fluorospheres (orange), β1 integrin (green), and actin (red). Scale bar = 100 μm (b,c) Zoomed in images of invasive cells (from the white box in (a)) showing cellular morphology and appropriate localization of (b) β1 integrin and (c) actin. Scale bar = 20 μm.

a

Technique	Imaging Context	Good Fits (% of total)
Widefield	High	0 (0%)
	Low	5 (2%)
	DISC-3D	194 (65%)
Confocal	High	55 (18%)
	Low	272 (91%)
	DISC-3D	254 (85%)
Airyscan	High	279 (93%)
	Low	299 (>99%)
	DISC-3D	293 (98%)
SIM	High	214 (71%)
	Low	296 (99%)
	DISC-3D	294 (98%)
STORM	High	No data
	Low	No data
	DISC-3D	235 (78%)

b

T test FWHM	High	Low
Widefield	No data	***
Confocal	***	†
Airyscan	***	***
SIM	***	†
STORM	No data	No data

T test Pore Size	High	Low
Widefield	No data	*
Confocal	**	***
Airyscan	**	***
SIM	†	†
STORM	No data	No data

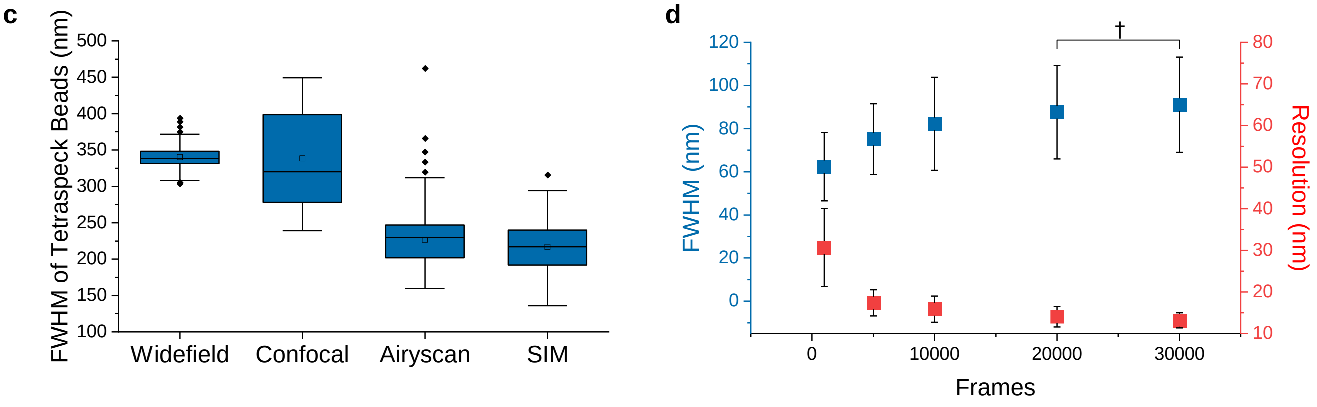

Figure S4. Details associated with fiber width determination. (a) Percent of visualized fibers in which an intensity line profile across the fiber resulted in a good fit ($R^2 > 0.95$) to a Gaussian across microscopy techniques and imaging context for images such as those shown in Fig. 4 in the Main Text. (b) Statistical table outlining the results of pairwise t-tests of (top) the full width at half maximum (FWHM) and (bottom) pore size between DISC-3D and images taken either high or low within the gel (data shown in Fig. 4b and c in the Main Text). “no data” indicates comparisons that were not possible because the technique did not provide adequate data for further analysis; * $p < 0.05$; ** $p < 0.01$; *** $p < 0.001$; † $p > 0.5$. (c) Microscope resolution determined via FWHM of 100 nm TetraSpeck beads (described in Methods) for all microscopic methods except STORM. (h) Determination of resolution limit as well as FWHM of measured collagen fibers as a function of STORM frames (described in Materials and Methods). The FWHM of fibers assessed by STORM here is somewhat larger than that in Fig. 4b in the Main Text because only fibers sufficiently thick to be characterized at 1000 frames could be included in this analysis.

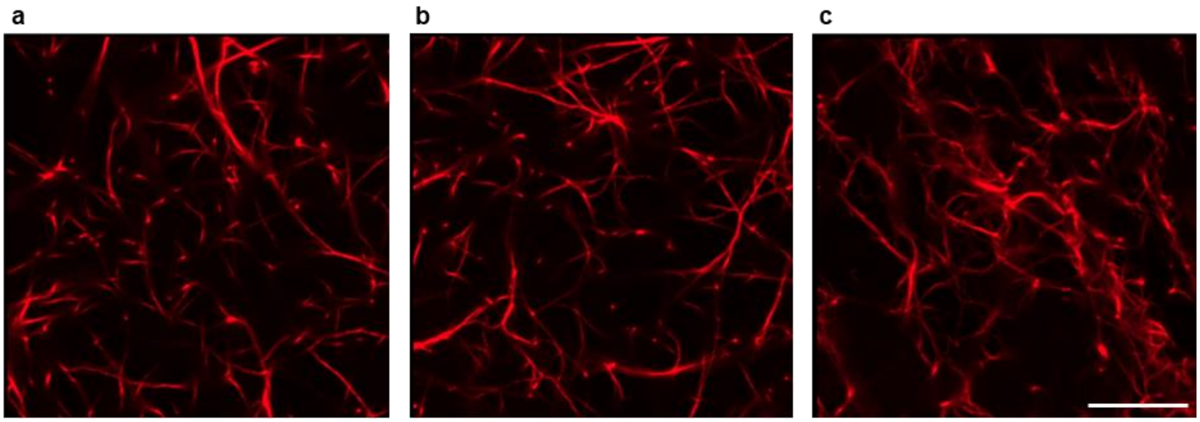

Figure S5. Representative images of a collagen I sample (a) following gelatin addition and gelation (equivalent to Fig. 4a, top row green image in the Main Text), (b) gelatin addition and gelation and flash freezing, and (c) gelatin addition, gelation, flash freezing, and removal of silicone spacers. All images are taken ≈ 10 μm above the coverslip with Airyscan imaging. Scale bar = 10 μm.

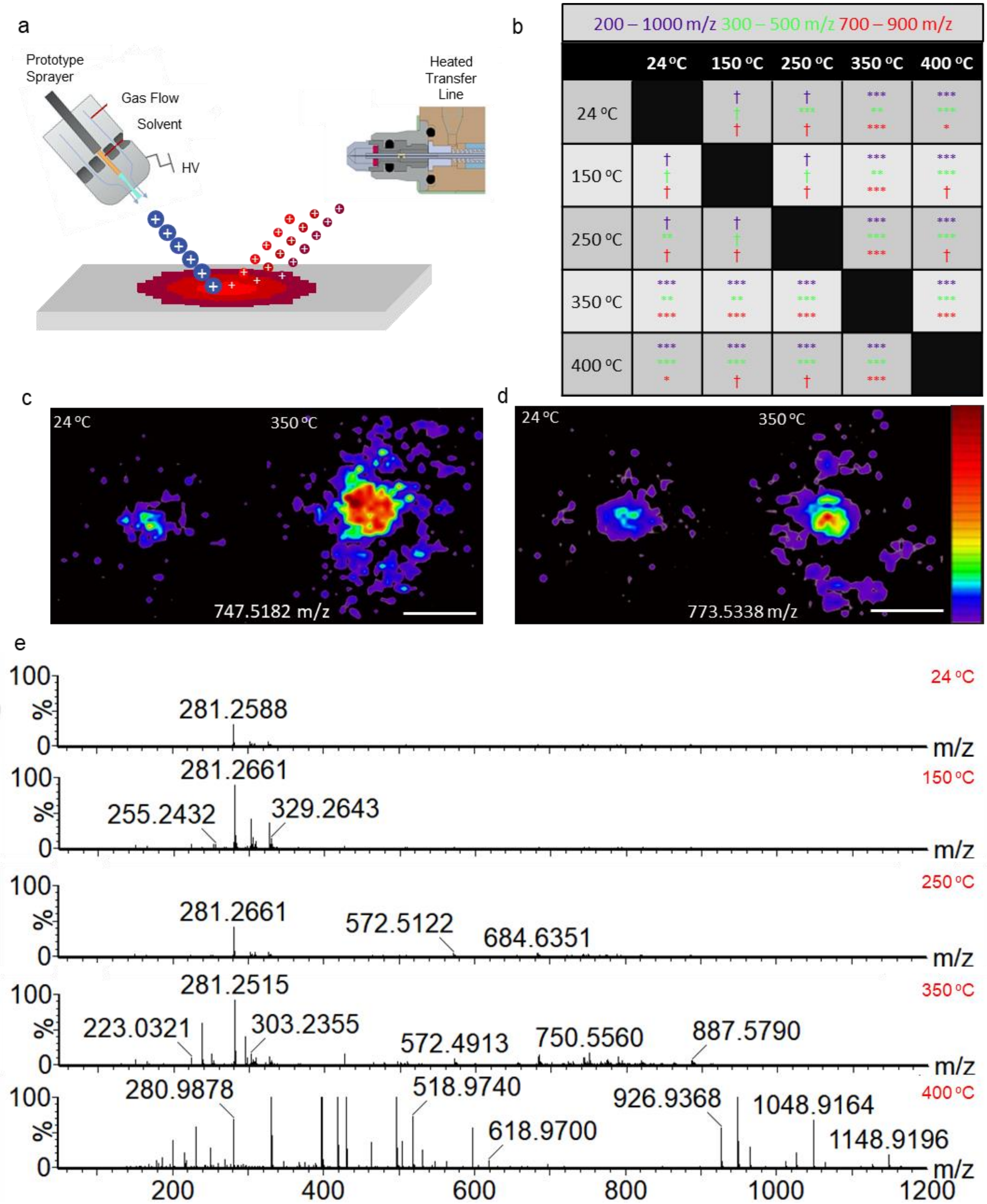

	24 °C	150 °C	250 °C	350 °C	400 °C
24 °C		† † †	† *** †	*** ** ***	*** *** *
150 °C	† † †		† † †	*** ** ***	*** *** †
250 °C	† ** †	† † †		*** *** ***	*** *** †
350 °C	*** ** ***	*** ** ***	*** *** ***		*** *** ***
400 °C	*** *** *	*** *** †	*** *** †	*** *** ***	

Figure S6. Details associated with mass spectrometry imaging measurements. (a) Depiction of the experimental setup for DESI-MSI. A cryosectioned sample (red) is placed on a glass slide. A solvent sprayer (medium grey) creates a solvent spray (blue) that, when combined with the nebulizing nitrogen gas (green arrows), creates charged solvent particles (blue spheres). These particles form a film atop the sample surface, interacting with the sample to form secondary ions comprised of the analyte (red spheres). The secondary ions are desorbed, ejected, and ionized. They enter the mass spectrometry atmospheric transfer line inlet, where they can be analyzed. The prototype sprayer has a pre-built emitter cartridge, creating a solvent spray with a finer radius that results in high spatial resolution. (b) Summary of pairwise t tests at temperatures from 24 ^{o}C to 400 ^{o}C and at m/z of 200 – 900 (purple), 300 – 500 (green), and 700 – 900 (red) (Full data set shown in Fig. 5a-c in the Main Text). (c,d) Representative MSI of spheroids taken at 24°C and 350°C of (c) glycerophosphoglycerol 34:1 (747.5182 m/z) and (d) glycerophosphoglycerol 36:2 (773.5338 m/z) showing the increase in signal, particularly in the invasive front, for both of these signals. Color scale is the same as in Fig. 5 in the main text, normalized to the maximum intensity of the image taken at the higher temperature. Scale bar = 500 μm. (e) Representative normalized mass spectra collected from invasive spheroids following the DISC-3D protocol at all temperatures investigated over the full m/z range demonstrating signal intensity in the ≈ 650 – 900 m/z regime is maximized at 350°C. Labeled peaks are chosen at random from among the strongest intensity signals at each temperature.

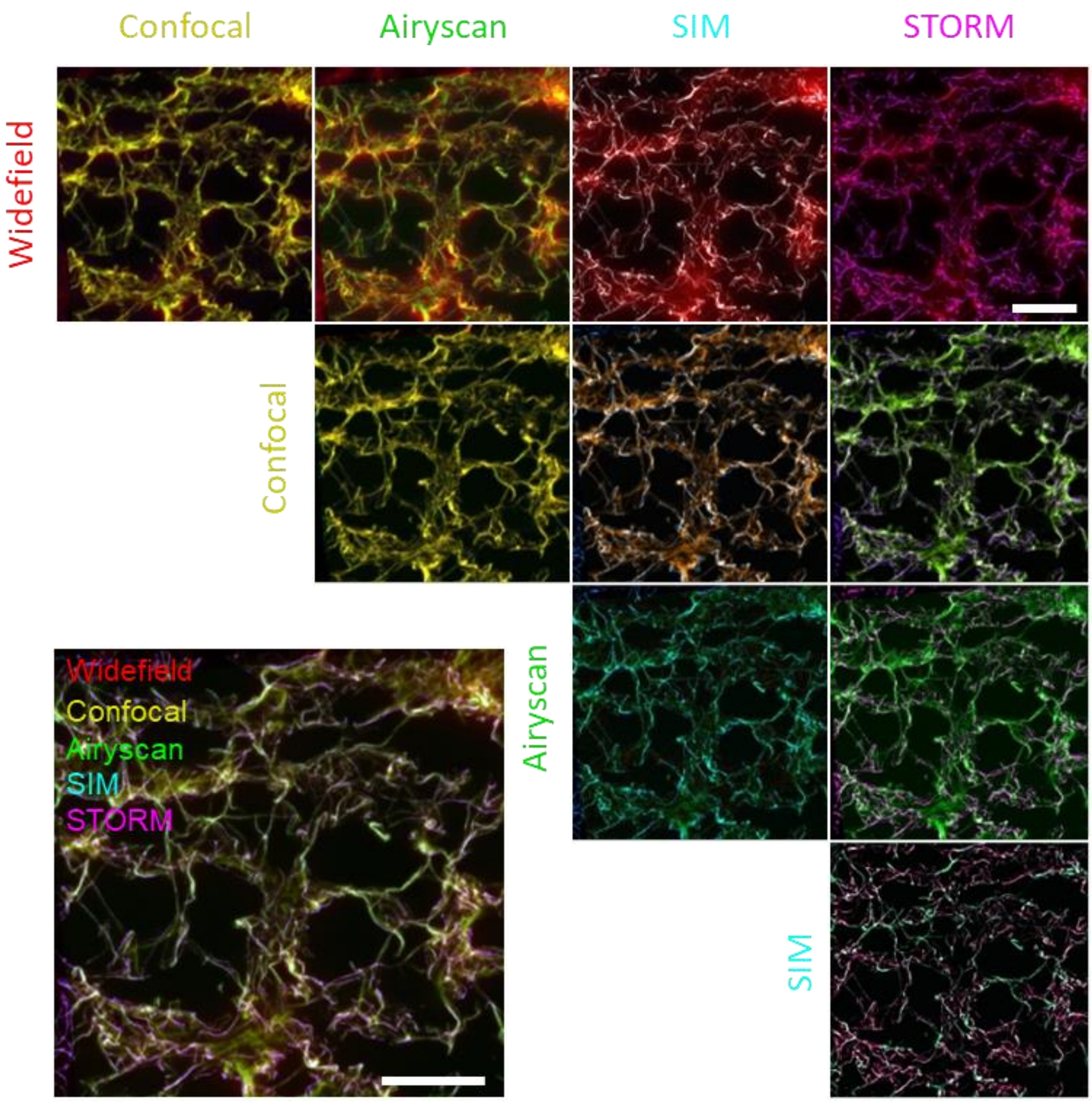

Figure S7. Correlative microscopy of DISC-3D prepared collagen I network using widefield microscopy, confocal microscopy, Airyscan imaging, lattice SIM, and STORM. Correlative images were generated using the TurboReg plugin in ImageJ. Images in the grid at right show all possible binary combinations of the aforementioned microscopy techniques. Image at the lower left shows all images overlaid. Contrast is not identical in image at lower left as in binary images. Scale bars = 10 μm.
